# Perivascular administration of metformin through 3D minichannels improved adventitia ingrowth and endothelialization for electrospinning vascular grafts

**DOI:** 10.1016/j.bioactmat.2026.07.027

**Published:** 2026-07-23

**Authors:** Yidong Hu, Zheqian Zhang, Pingping Yuan, Gaoyue Yang, Yuxin Lu, Xinchi Zhang, Xiaopeng Wu, Yajing Zhao, Pengyu Wang, Yujiao Wang, Ning Leng, Wenyu Zhan, Wei Wu

**Affiliations:** a State Key Laboratory of Oral & Maxillofacial Reconstruction and Regeneration, National Clinical Research Center for Oral Diseases, Shaanxi Key Laboratory of Stomatology, Department of Oral & Maxillofacial Surgery, School of Stomatology, The Fourth Military Medical University, Xi'an, 710032, China; b School of Pharmacy, Shaanxi University of Chinese Medicine, Xianyang, 712046, China; c State Key Laboratory of Oral & Maxillofacial Reconstruction and Regeneration, National Clinical Research Center for Oral Diseases, Shaanxi Key Laboratory of Stomatology, Department of Prosthodontics, School of Stomatology, The Fourth Military Medical University, Xi'an, 710032, China

**Keywords:** Vascular graft, 3D printing, Carotid artery reconstruction, Perivascular drug administration, Endothelialization

## Abstract

Achieving rapid endothelialization to maintain the patency of small-diameter vascular grafts remains a significant clinical challenge. Conventional surface modification strategies provide limited biochemical signals for recruiting circulating endothelial cells and fail to mitigate perivascular scarring and fibrosis. Emerging evidence underscores the critical role of regenerated adventitia in facilitating fallout endothelialization. Based on this rationale, we hypothesized that pharmacological administration around vascular grafts could promote rapid adventitia ingrowth and accelerate fallout endothelialization, therefore fabricated three-dimensional minichannel-wrapped vascular grafts loaded with collagen-lyophilized metformin sponges (DMwVGs), which facilitated sustained release of metformin (15.5% release at 72 h, sustained over 21 days) and promoted robust adventitia ingrowth. The patency rate of DMwVGs reached 83% (15/18 overall) in a rabbit carotid artery model at two weeks, significantly outperforming metformin-loaded electrospinning-only grafts (33%, 2/6). The DMwVGs facilitated the transmural growth of vascularized neo-adventitia, driving efficient fallout endothelialization. Integrated multi-omics assays revealed that DMwVGs promoted adventitia angiogenesis via activation of the MAPK pathway (2.4-fold increase) and inhibition of the NF-κB/TNF-α pathway (62% reduction). This study represents an innovative shift from passive luminal coatings to active perivascular regenerative therapy, offering a promising platform for engineering durable small-diameter vascular grafts.

## Highlights

• A 3D minichannel vascular graft enables sustained perivascular metformin delivery, promoting rapid adventitia ingrowth and accelerating fallout endothelialization in a rabbit carotid model.
• Transmural growth of vascularized neoadventitia serves as an active cellular reservoir that recruits circulating CD93^+^/CD31^+^ endothelial cells; multi-omics and functional assays identify CXADR-MAPK activation and NF-κB/TNF-α suppression as key mechanisms.
• Long-term (36-week) follow-up confirms stable trilayered vessel architecture, functional ECM remodeling, and partial reinnervation, demonstrating a shift from passive coatings to regenerative vascular grafts.

## Graphical abstract

**Schematic of the preparation of minichannel-wrapped vascular grafts(MwVGs) and the remodeling process in rabbit carotid arteries.** MwVGs were fabricated using a sacrificial caramel template strategy to create a minichannel external scaffold, complemented by an internal ultrathin electrospinning layer. During the remodeling process in rabbit carotid arteries, the minichannels facilitated sustained drug release. In the early post-implantation phase, DTβ4 mediated antithrombotic effects, followed by metformin, which promoted the transmural growth of vascularized neo-adventitia, thereby enhancing rapid endothelialization.

**Figure:**
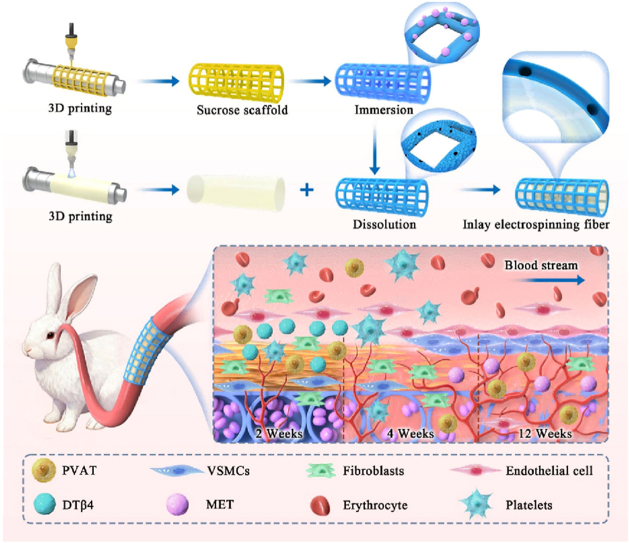

## 1 Introduction

Arterial reconstruction is critical for addressing clinical challenges such as war trauma, tumor resections, and vascular malformations [1]. Currently, large-diameter vascular grafts (diameter >6 mm), typically made from materials such as expanded polytetrafluoroethylene (ePTFE) and polyethylene terephthalate (PET), have demonstrated clinical effectiveness [2]. In contrast, small-diameter artificial grafts (diameter ≤6 mm) often exhibit higher failure rates. Statistical data has revealed that the patency rate is only 42% at twelve months after limb arteriovenous graft implantation in patients with end-stage renal disease [3]. Conventional postoperative management typically involves systemic antithrombotic therapy to maintain durable patency. However, studies have demonstrated that inadequate endothelialization in heparin-mediated dyslipoproteinemia can contribute to progressive intimal hyperplasia [4], which is particularly pronounced in small-diameter artificial blood vessels. Consequently, addressing the challenges associated with inherent anticoagulation represents a critical scientific endeavor in the development of small-diameter vascular grafts.

Recent advances in tissue-engineered vascular grafts (TEVGs), particularly through surface modifications using hydrophilic polyethylene glycol, zwitterionic polymers, heparin, and other bioactive molecules, have significantly enhanced their antithrombotic properties. Studies have demonstrated that the heparin-coated inner layer derived from extracellular matrix (ECM) significantly improves hemocompatibility [5,6]. More recently, reinforced biotubes have been developed as readily available and regenerative vascular grafts with favorable mechanical and immunomodulatory properties [7]. Furthermore, tubular polycaprolactone (PCL) grafts modified via electrospinning and coated with Nap-FFRGD effectively inhibit platelet adhesion [8]. Although surface modifications address short-term thrombosis, they are inherently limited by insufficient drug-loading capacity and poorly controlled release kinetics under hemodynamic shear stress [[9], [10], [11], [12]]. Beyond immediate antithrombotic effects, achieving long-term patency requires sustainable re-endothelialization. Theoretically, synthetic vascular grafts achieve endothelialization through endothelial cell pre-seeding, transanastomotic ingrowth, and the recruitment of circulating endothelial progenitor cells (EPCs) [13,14]. Due to the shear stress exerted by the bloodstream, the homing process driven by EPCs is crucial for bridging the period between anticoagulation and endothelialization. Notably, the recruitment of circulating EPCs is partially regulated by signals from vascular precursor cells located in the adventitia and perivascular tissue (PVT). Yuan et al. fabricated electrospun vascular grafts with a macro-porous outer layer, which facilitated adventitial tissue integration and thereby promoted rapid endothelialization [15]. Our previous study illustrated the cellular heterogeneity in the regenerated adventitia and intima of 3D-printed hierarchical vascular grafts through spatial transcriptomics [16]. Furthermore, we demonstrated that micro-nano structured vascular grafts achieved rapid endothelialization as early as two weeks, underscoring the significance of adventitia angiogenesis in promoting endothelialization. These findings highlight the pivotal role of adventitia ingrowth in promoting rapid endothelialization during the host remodeling of vascular grafts [17]. Despite of these progresses, most existing strategies for drug administration suffered from low drug-loading capacity and uncontrolled burst release [18]. Furthermore, no study has yet exploited the perivascular space as a sustained drug delivery reservoir to actively promote adventitia regeneration and subsequent fallout endothelialization. The adventitia is increasingly recognized as an active regenerative niche, but pharmacological approaches to target this niche in a controlled, durable manner have not been developed for small-diameter vascular grafts.

Sacrificial biomanufacturing has emerged as an innovative strategy for engineering minichannels, effectively bridging the gap between structural fidelity and functional complexity. This technique facilitates the fabrication of minichannel constructs that accurately replicate the structural gradients observed in native tissues, with broad applications across various fields, including cardiac, hepatic, and bone regeneration [[19], [20], [21], [22]]. Previous study demonstrated that collagen-metformin lyophilized powder significantly improved the adventitia regeneration in the “perivascular tissue deprived” vein grafting model, which inspired us to accelerate adventitia regeneration through perivascular sustained-release metformin, thereby stimulating the recruitment of circulating endothelial cells [23]. Consequently, aiming at durable perivascular drug administration, we constructed 3D porous minichannels utilizing sacrificial caramel templating. This 3D perfusable minichannel system served not only as a high-capacity reservoir but also as a platform for the stable and sustained release of metformin, which continuously promoted transmural growth of vascularized neo-adventitia, thereby accelerating fallout endothelialization. Overall, we present a 3D minichannel-wrapped vascular graft that employs perivascular drug administration to facilitate rapid endothelialization via accelerated adventitia angiogenesis, thereby directly addressing the identified gap by enabling sustained, high-capacity perivascular drug delivery to harness the regenerative potential of the adventitia, which represents an innovative shift from conventional lumen surface modification strategies.

## 2 Results

### 2.1 Fabrication of 3D minichannel-wrapped vascular grafts

**Fig. 1:**
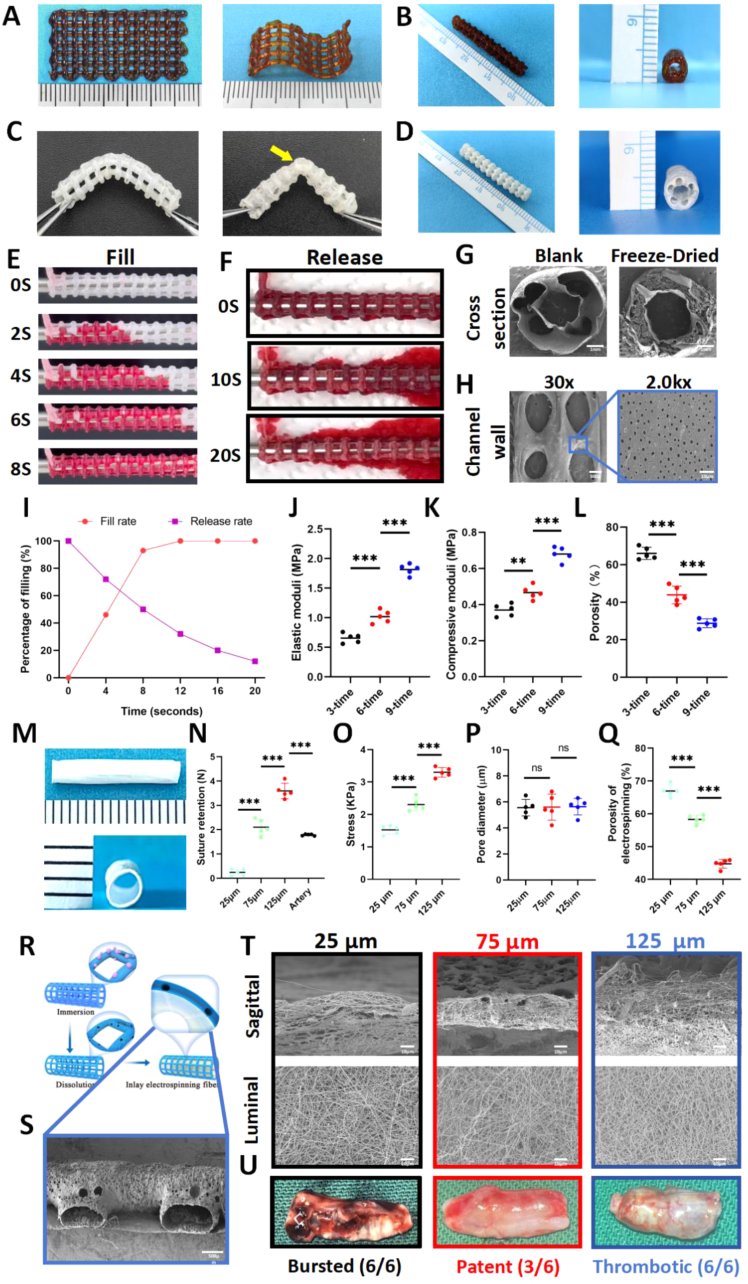
**Preparation and Characterization of MwVGs and ES.** (A) Preparation of highly ductile caramel models. (B) Preparation of caramel sizes tailored for carotid artery transplantation in rabbits. (C) MS exhibited flexibility. (Yellow arrow: stents composed of 80% PCL and 20% PGS induce clamping.(D) MS suitable for rabbit carotid artery transplantation. (E-F) The distribution of red ink in MS and its permeation on absorbent paper. (G) Freeze-dried collagen was fully and evenly distributed within MS. (H) Porous tube wall structures of MS. (I) Quantitative characterization of red ink perfusion and sustained release. (J-K) Compressive and elastic moduli of MS with different coating times. (L) Characterization of porosity across various coating times. (M) The gross morphology of the ES. (N-O) Mechanical properties of the ES were shown by suture retention strength (N) and burst pressure (O). (P) Pore diameter of ES (n = 5 in each group). (Q) Porosity of the ES. (R-S) Scheme and scanning electron microscopy image of MwVGs. (T) Scanning electron microscopy image of ES with different thicknesses. (U) The gross morphology of various ES at 2 weeks post-implantation. For (J-L, N-Q), significance was determined by one-way ANOVA followed by Tukey's post hoc analysis (n = 5 independent samples). Plotted data represent means ± SD. **: P < 0.01, ***: P < 0.001.

Tubular caramel templates matching the anatomical dimensions of the rabbit carotid artery were fabricated (Fig. 1A and B). Using a highly ductile sacrificial caramel template (Fig. 1A) combined with a coating phase separation technique, we fabricated a three-dimensional interconnected minichannel scaffold (MS) coated with a blend of poly (glycerol sebacate) (PGS) and PCL. The incorporation of PGS not only accelerates the degradation of the hybrid coating but also imparts flexibility. In contrast, a pure PCL-coated MS exhibited reduced flexibility and was prone to clamping during bending (Fig. 1C). Consistent with our previous study [16], a blending ratio of 20% PGS/PCL achieved an optimal balance between degradation rate and elastic recovery, meeting the mechanical requirements for vascular grafts (Fig. 1D). Mechanical strength increased with the number of coating times; however, this was accompanied by a progressive reduction in porosity and distinct microstructural alterations. Scanning electron microscopy (SEM) images revealed that the 3-time coating MS exhibited collapsed channels, while the 9-time coating MS was excessively thick and demonstrated the lowest porosity (Fig. S1A). Quantitative analysis showed that increasing coating times elevated both the elastic modulus (3-time coating: 0.66 ± 0.08 MPa; 6-time coating: 1.02 ± 0.11 MPa; 9-time coating: 1.81 ± 0.09 MPa) and the compressive modulus (3-time coating: 0.37 ± 0.03 MPa; 6-time coating: 0.47 ± 0.04 MPa; 9-time coating: 0.68 ± 0.04 MPa) (Figures S1B-C, Fig. 1J–K), while significantly reducing overall porosity (3-time coating: 66.64 ± 2.97%; 6-time coating: 43.88 ± 4.67%; 9-time coating: 28.78 ± 2.36%) (Fig. 1L). The 6-time coating provided a balance: elastic modulus 1.02 MPa, compressive modulus 0.47 MPa, and porosity 43.9% with interconnected channels. Based on these findings, the 6-time coating MS was selected as the optimal balance, providing adequate mechanical strength while maintaining suitable porosity. Utilizing these parameters, we fabricated an MS with an inner diameter of 1.98 ± 0.21 mm and a minichannel diameter of 0.93 ± 0.08 mm (Fig. 1D).

The minichannel scaffold exhibited high fluid permeability and exceptional drug-loading capacity. Perfusion experiments confirmed that the network could be fully filled with red ink within 20 s, suggesting high patency and low flow resistance (Fig. 1E–F and 1I). Notably, the hollow architecture of MS provided an ideal platform for high-concentration drug loading. To achieve this, we utilized collagen solution as a drug carrier, which forms a homogeneous dispersion with hydrophilic drugs in its liquid state. During the lyophilization process, an ice-templating effect generated a highly porous three-dimensional network that effectively encapsulated drug molecules while establishing sustained release [24,25]. This collagen-based lyophilization facilitated efficient drug encapsulation within the minichannels. SEM confirmed that the lyophilized collagen matrix completely occupied the internal minichannel space, resulting in a uniformly porous structure (Fig. 1G). The distinctive absorption bands of metformin in DMwVGs were recognized through Fourier transform infrared (FTIR) spectroscopy (Fig. S1D). The drug release profile was critically regulated by the microstructure of the collagen matrix. By incorporating NaCl as a porogen, we engineered microporous channel walls to modulate drug diffusion (Fig. 1H). Release kinetics were evaluated in a Matrigel system using Cyanine3-labeled metformin (Cy3-MET) as a fluorescent tracer. Fluorescence imaging and quantification revealed a sustained diffusion profile: the diffusion front advanced from 350.1 ± 17.05 μm at 24 h to 660.1 ± 21.09 μm at 48 h, with fluorescence intensity increasing gradually at all measured points, confirming controlled release from the minichannels (Fig. S1J–L).

To fabricate the inner layer of the minichannel-wrapped vascular grafts (MwVGs), we employed electrospinning of PCL, which was chosen for its mechanical properties and biocompatibility (Fig. 1M). To identify the optimal inner layer thickness, we fabricated PCL layers with wall thicknesses of 25 μm, 75 μm, and 125 μm by adjusting electrospinning duration and systematically evaluated their key properties for implantation. Mechanical evaluation revealed a clear dependence on thickness. Suture retention strengths for the three groups were 0.24 ± 0.13 N, 2.10 ± 0.33 N, and 3.60 ± 0.32 N, respectively (Fig. 1N). Notably, the 75 μm graft exhibited a higher suture retention strength than that of the human internal mammary artery (1.40 ± 0.01 N) and the human saphenous vein (1.81 ± 0.02 N, although it was lower than the breaking force of the 7-0 polypropylene suture (3.7 N) [26], thus fully satisfying the surgical requirements for vascular replacement procedures. Burst pressure examination demonstrated a clear correlation between burst pressure and thickness of electrospun PCL layer (Fig. 1O). The 25 μm group exhibited insufficient strength (1.53 ± 0.14 × 10^3^ mmHg), resulting in post-implantation rupture. Conversely, the 125 μm group showed high burst strength (3.30 ± 1.48 × 10^3^ mmHg) but resulted in complete coagulation within two weeks. Although no significant difference was observed in the pore diameter (Fig. 1P), the porosity of the electrospun PCL fibers decreased with increasing thickness (25 μm: 66.96 ± 1.79%, 75 μm: 58.28 ± 1.26%, and 125 μm: 44.76 ± 1.34%) (Fig. 1Q). Overall, the 75 μm PCL layer was selected due to its optimal balance between burst strength and porosity.

To demonstrate the advantage of the perivascular minichannels over metformin-loaded electrospinning strategy, we prepared control grafts in which metformin was loaded onto PCL fibers via electrospinning (DES). Compared with DES, metformin-loaded MwVGs (DMwVGs) enhanced the loading capacity per centimeter by 4.4-fold, effectively overcoming the inherent loading limitations of electrospinning (Fig. S1E). More critically, the release profiles differed substantially. ELISA analysis revealed that DMwVGs exhibited favorable release kinetics, with an initial release of approximately 15.5 ± 2.4% within 72 h, followed by a prolonged release for over 21 days. In contrast, the DES group showed a significant burst release (80.3 ± 5.7% within 72 h), which rapidly declined and was nearly complete (96.3 ± 2.3%) by day 7 (Fig. S1F).

To quantify the absolute drug loading and release kinetics, we performed LC-MS/MS analysis under physiologically relevant conditions (PBS containing 0.1% BSA and 0.01% collagenase, with orbital shaking at 60 rpm). Absolute metformin loaded per cm graft in DMwVGs was 343.8 ± 10.8 μg, which was 4.4-fold higher than that of DES (77.3 ± 0.4 μg) (Fig. S1I). The absolute cumulative release profile revealed that DMwVGs released 128.5 ± 8.7 μg per cm graft by day 21, maintaining a sustained release over the entire observation period. In contrast, DES nearly exhausted its drug content by day 7 (74.5 ± 2.1 μg per cm graft) (Fig. S1G). To validate whether the *in vitro* release translated into effective local drug accumulation, we harvested perivascular tissues at serial time points and measured metformin concentration via LC-MS/MS. In the DMwVG group, the local tissue concentration remained stable at 11.9 ± 1.4 μg/g at day 15, which is within the therapeutic range reported in the literature [27]. However in the DES group, the concentration peaked at 2.9 ± 0.5 μg/g at day 3 and declined thereafter (Fig. S1H). Linear regression analysis further established a strong positive *in vitro*-*in vivo* correlation (IVIVC) for DMwVGs (r = 0.924, R^2^ = 0.854), in stark contrast to the negative correlation observed in the DES group (r = −0.765) (Table S3). These data confirmed that the minichannel-collagen system achieved sustained perivascular metformin delivery with effective local tissue accumulation over the critical early remodeling window.

### 2.2 Angiogenic, antithrombogenic properties and *in vitro* cytocompatibility of DMwVGs

**Fig. 2:**
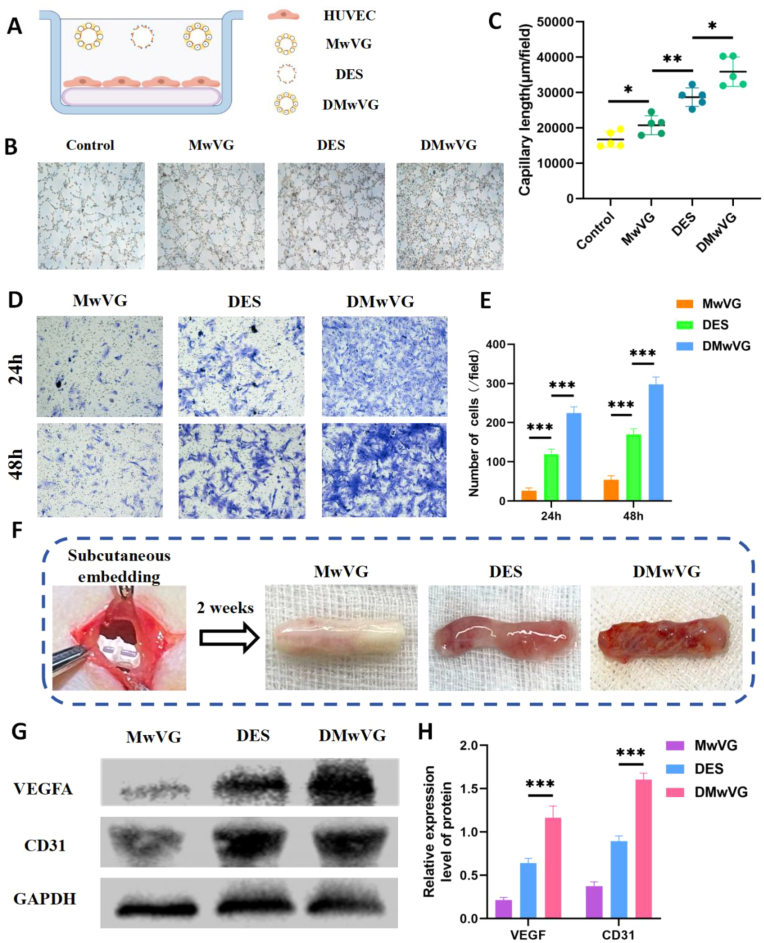
**The angiogenic properties of DMwVGs was evaluated *in vivo* and *in vitro*.** (A and B) A tube formation assay was utilized to demonstrate the influence of MwVGs and ES on endothelial cell function. (C) The total tube length was analyzed using ImageJ. (D) Transwell assays were conducted for different grafts. (E) Quantitative analysis of HUVECs across the three groups at 24 and 48 h was performed. (F) Gross views of the grafts were subcutaneous embedded for 2 weeks (n = 5 in each group). (G and H) Western blot analyses for VEGFA and CD31 after 2 weeks (n = 5 independent samples). For (C, F, H), significance was determined using one-way ANOVA followed by Tukey's post hoc analysis. The plotted data represent means ± SD. ***: P < 0.001.

The sustained release of metformin in DMwVGs accelerated tissue regeneration and enhanced migration and recruitment of endothelial cells. Tube formation experiments demonstrated that the tubulogenic capacity of human umbilical vein endothelial cells (HUVECs) was markedly increased when treated with DMwVGs leaching solution compared to the DES group (Fig. 2A–C). The pro-migratory effect on endothelial cells was further validated using a transwell assay. After 24 h, cell migration in the DMwVGs group (224.6 ± 15.5 cells/field) significantly exceeded that in the DES (119.4 ± 12.7 cells/field) and MwVGs (26.2 ± 7.4 cells/field) groups (p < 0.01). This difference became more pronounced at 48 h (DMwVGs: 298.2 ± 18.4; DES: 170.6 ± 13.9; MwVG: 54.0 ± 10.4) (Fig. 2D and E). Notably, while the DES group showed an initial pro-migratory effect at 24 h, its efficacy diminished by 48 h, correlating with the burst release and subsequent rapid decline of electrospinning layer. In contrast, the DMwVGs group maintained a stable and enhanced effect over time, underscoring the advantage of sustained release from the minichannels. The pro-angiogenic efficacy was further confirmed *in vivo*. After two weeks of subcutaneous embedding, the DMwVGs surface was covered with notably more abundant capillary networks compared to the sparse angiogenesis observed on MwVGs (Fig. 2F). Mechanistically, Western blot analysis for the perivascular tissue indicated that the sustained metformin release enhanced angiogenesis by upregulating the expression of CD31 and VEGFA (Fig. 2G and H). Collectively, these findings confirmed that the minichannel-based sustained delivery platform enhanced bioactivity compared to the DES group.

The incorporation of DTβ4 into PCL electrospinning rendered MwVGs antithrombogenic. Vascular grafts of ES + DTβ4 were implanted in rabbit carotid artery models and subsequently explanted after 2 h. Patency was confirmed in all cases (5/5) through distal arterial refilling tests (Fig. S3A). Additionally, the antithrombogenic properties of the ES + DTβ4 membranes were validated *in vitro* by incubating with recalcified whole blood (Fig. S3B–F).

The cytocompatibility of the DMwVG was assessed using HUVECs. Immunofluorescence and SEM analyses demonstrated that HUVECs formed a continuous monolayer with a well-spread morphology and an organized actin cytoskeleton, indicating excellent biocompatibility (Fig. S2A and B). Cells exhibited favorable adhesion on day 1, and proliferation significantly increased from 0.73 × 10^4^ to 3.69 × 10^4^ over 7 days, reflecting a 4.05-fold increase (Fig. S2C and D). Based on the experimental findings, DMwVGs demonstrated multifunctional advantages: it promoted angiogenesis through sustained metformin release, provided effective antithrombogenic properties and exhibited excellent cytocompatibility.

### 2.3 DMwVGs maintained high patency rates and promoted neo-adventitia ingrowth

**Fig. 3:**
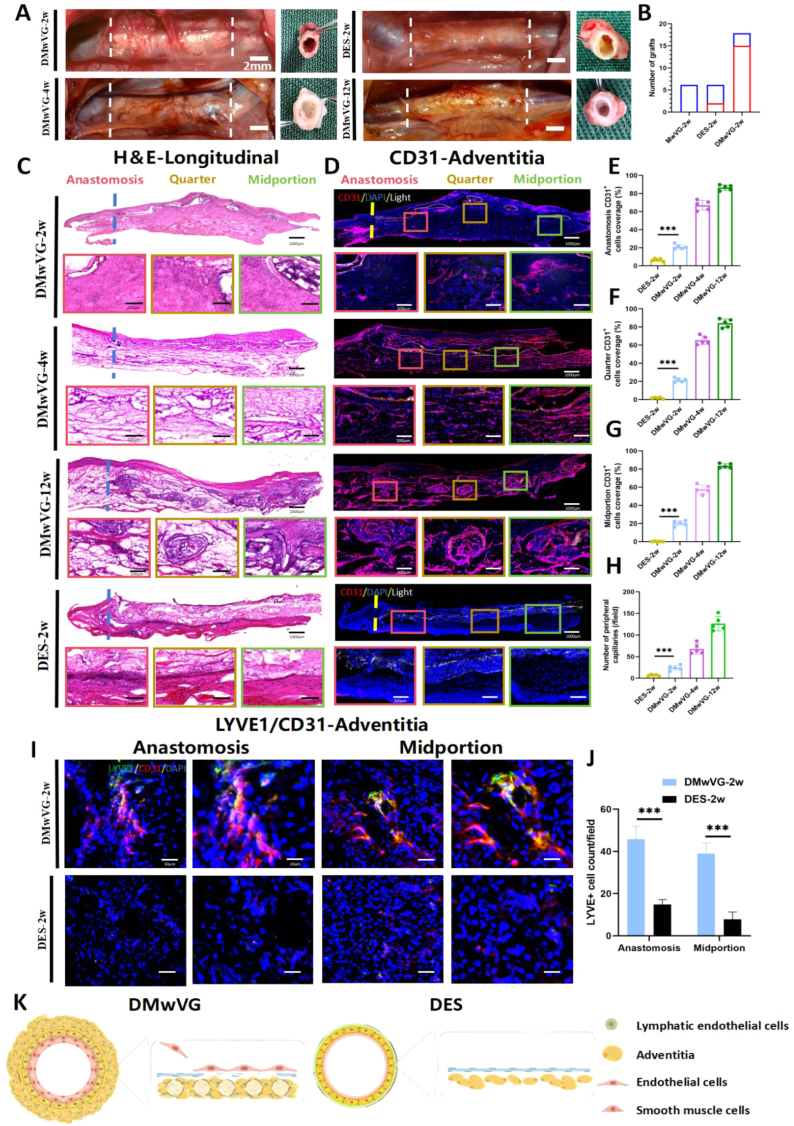
**The gross morphology and adventitia ingrowth of DES and DMwVGs over 12 weeks.** (A) The gross morphology of the MwVGs is presented at 2, 4, and 12 weeks post-implantation, with the anastomosis sites indicated by the white dotted line. (B) The number of patent animals at 2 weeks is quantified. (C) H&E staining of longitudinal sections at each time point. (D) Longitudinal sections of CD31^+^ endothelial cells in neoarteries. (E-G) Quantification of endothelial coverage was performed by calculating the percentage of the CD31^+^ positive area relative to the total luminal area at the suture, quarter, and middle sites at each time point (n = 5 independent samples). (H) The number of peripheral capillaries was assessed at each time point (n = 5 independent samples). (I) Immunofluorescence staining of LYVE1+ cells. (J) Count of LYVE1+ cells in adventitia (n = 5 independent samples). (K) Schematic illustration of the adventitial and endothelialization induced by different drug loading structures. For (E-H), statistical significance was determined using one-way ANOVA followed by Tukey's post hoc analysis. For (J), significance was determined by Student's t-test. ***: P < 0.001.

To evaluate the *in vivo* performance of DMwVGs in arterial circulation, we implanted DMwVGs (n = 18) to replace the left carotid artery in rabbits. The metformin-loaded electrospinning grafts (DES) served as a control group (n = 6), while the MwVG group (n = 6) acted as an additional control. Following the implantation of 1.5 cm long grafts with one week systemic anticoagulation, we conducted a comprehensive assessment of vascular remodeling at specific intervals (2, 4, and 12 weeks) (Fig. 3A). Sustained patency of DMwVGs was accompanied by robust perivascular tissue regeneration and integration. Notably, abundant neovascularization encapsulating the scaffold was observed during the early stage at 2 weeks post-implantation, with the perivascular tissue becoming increasingly dense and organized over 12 weeks. At 2 weeks, the patency rate was 83% (15/18). Critically, all 15 patent grafts remained patent until their scheduled endpoints (4 or 12 weeks). In stark contrast, the DES group showed only 33% patency (2/6) at 2 weeks, and the MwVGs group was completely coagulated (0/6) by this time point (Fig. 3B). Hemodynamic function in patent DMwVGs was comparable to native arteries. Long-term Doppler ultrasound monitoring revealed no significant differences in hemodynamic parameters (Fig. S4A–C) (peak systolic velocity: 2 weeks: 90.4 ± 2.6 cm/s, 4 weeks: 92.5 ± 6.9 cm/s, 12 weeks: 93.2 ± 3.3 cm/s, Native: 85.8 ± 3.3 cm/s) between DMwVGs and the native artery at any time point. Conversely, blood flow in the patent DES grafts was markedly compromised (44.9 ± 3.9 cm/s at 2 weeks) due to mural thrombus formation (Fig. S4A–C).

Hematoxylin and eosin (H&E) and immunofluorescence staining analyses revealed rapid ingrowth and angiogenesis of the adventitia in DMwVGs. At 2 weeks after implantation, DMwVGs exhibited robust neovascularization, demonstrating complete integration with the perivascular tissue (Fig. 3C). Notably, CD31 immunofluorescence staining confirmed a progressive increase in CD31^+^ cells within DMwVGs, covering the anastomosis, quarter and midportion. Quantifications presented a significant increase in CD31^+^ cells from 2 weeks to 12 weeks (2 weeks: 20.53 ± 1.70%; 4 weeks: 60.43 ± 3.18%; 12 weeks: 92.67 ± 3.61%) (Fig. 3E–G), which was the evidence for the angiogenesis in the neo-adventitia. In stark contrast, the DES group exhibited poor integration with the perivascular tissue, characterized by sparse peripheral capillary formation and encapsulation by a thin fibrous layer (Fig. 3C–G). Quantitative assessments of CD31^+^ cells in the neo-adventitia of the DES group were significantly lower than those in DMwVGs from 2 weeks to 4 weeks (2 weeks: 1.67 ± 0.31; 4 weeks: 2.98 ± 0.51). These results indicate notable differences in the cellular composition of the neo-adventitia between the two groups.

Our previous studies have demonstrated that lymphatic endothelial cells (LECs) enriched in the neo-adventitia, marked by LYVE1, were identified as the main contributors to the recruitment of fallout endothelialization [17]. To further investigate the impact of LYVE1^+^ endothelial cells, en-face staining was conducted on the neo-adventitia. The counts of LYVE1^+^ cells in all neo-adventitia locations within the DMwVGs (anastomosis, 45.8 ± 6.0 cells per field; midpoint, 39.0 ± 5.0 cells per field) exceeded those observed in the DES group (anastomosis, 14.8 ± 2.4 cells per field; midpoint, 7.8 ± 3.5 cells per field). (Fig. 3I and J). Collectively, these data demonstrated that DMwVGs facilitated not only high patency but also transmural growth of vascularized neo-adventitia, significantly outperforming DES.

### 2.4 Transmural growth of vascularized neo-adventitia in 3D minichannels of DMwVG promoted fallout endothelialization and intima regeneration

**Fig. 4:**
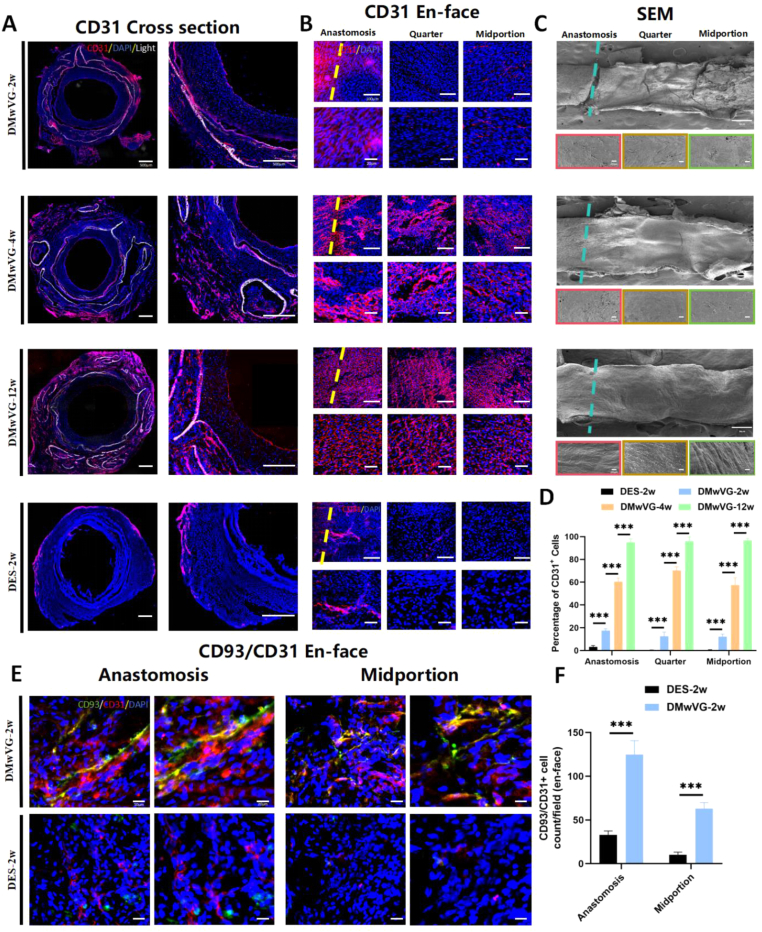
**Progressive endothelialization of DMwVGs was observed within 12 weeks.** (A) CD31 immunofluorescence staining of cross-sections at the middle sites of DES and DMwVGs (yellow dotted line indicated the suture sites). (B) En-face analysis of CD31**^*+*^** cells at the anastomosis, quarter, and mid-portion of neoarteries. (C) SEM examination for luminal morphology of neoarteries at each time point (blue dotted line indicated the suture sites). (D) The number of CD31**^*+*^** cells at the middle sites of DES and DMwVGs was quantified at each time point (n = 5 independent samples). (E-F) En-face analysis and quantification of CD93**^*+*^**CD31**^*+*^** cells at the anastomosis and mid-portion of neoarteries (n = 5 independent samples). For (D), statistical significance was determined using one-way ANOVA followed by Tukey's post hoc analysis. For (F), significance was determined by Student's t-test. ***: P < 0.001.

Luminal endothelialization is critical for reducing thrombus formation and platelet activation, thereby maintaining normal blood flow and ensuring long-term vascular patency. To visualize the endothelialization process of neoarteries, longitudinal sections were obtained at each time point for CD31 immunofluorescence staining. CD31^+^ cells predominantly covered the suture site, with several cells appearing in the quarter and midportion of the grafts at 2 weeks. The observed discontinuous distribution pattern of CD31^+^ cells suggested two potential sources of endothelial cells: those migrating transmurally from perivascular tissues and circulating EPCs derived from the bloodstream, rather than transanastomotic ingrowth. These cells progressively increased in number and covered most of the luminal side after 12 weeks. Spatially, CD31^+^ cells were preferentially concentrated around the minichannels at 2 weeks post-implantation and subsequently infiltrated the adventitia of the neoartery by 4 weeks (Fig. 3G and 4A). Quantitative analyses revealed a significant increase in angiogenesis within the adventitia from 4 weeks to 12 weeks (2 weeks: 1.53 ± 0.39; 4 weeks: 2.98 ± 0.51; 12 weeks: 7.51 ± 1.13) (Fig. 3H–K). Notably, adventitia angiogenesis preferentially localized in the abluminal region of the minichannels in the graft wall rather than on the neointimal side. These observed spatiotemporal differences between luminal and abluminal endothelial cells suggested distinct cellular origins for endothelial cells in these two compartments, which aligned with current literature on fallout endothelialization [16]. In contrast, the DES group showed significantly fewer CD31^+^ cells, as confirmed by reduced ECs density throughout the graft length (Fig. 4A–D).

Multiple imaging techniques were performed to gain further insight into the involvement of luminal endothelialization. En face immunostaining revealed that ECs migrated from host artery and covered anastomosis gap at 2 weeks (Fig. 4B–D). Additionally, the ECs appeared as multiple clusters at the mid-graft and quarter-point regions, rather than progressing linearly from the anastomotic sites. At 4 weeks, the migration distance of ECs at the anastomosis sites remained limited, as evidenced by the discontinuities in the endothelial lining between the anastomosis and quarter sites. However, the number of CD31^+^ cells significantly increased at the mid and quarter points of the neoarteries. By 12 weeks, the increasing ECs progressively merged and nearly covered the entire luminal surface (Fig. 4B–D). Compared with the DMwVGs group, DES group exhibited significantly fewer endothelial cells on the luminal side, with markedly reduced endothelial cell density throughout graft wall (Fig. 4B–D). Concurrently, SEM provided ultrastructural evidence of progressive endothelial maturation in DMwVGs. At 2 weeks, the luminal surface was populated by spindle-shaped cells aligned with the direction of blood flow. By 4 weeks, co-deposition of cells and ECM had formed an immature yet continuous tissue layer. At 12 weeks, the lumen exhibited a mature endothelial phenotype, characterized by complex micro-fold structures, distinct cell borders, and mature cell-cell junctions, closely resembling native arteries (Fig. 4C). Our previous studies have demonstrated that circulating CD93^+^ endothelial cells play a crucial role in fallout endothelialization [17,28]. To further investigate the contribution of these cells to early graft endothelialization, en-face staining was performed on the neoendothelium at 2 weeks. In the DMwVG group, CD31^+^/CD93^+^ endothelial cells were observed at both the anastomosis (126.4 ± 21.7 cells/field) and the mid-graft (64.0 ± 13.2 cells/field). In contrast, the DES group exhibited markedly reduced numbers of CD31^+^/CD93^+^ cells at all neointimal sites (anastomosis: 34.0 ± 8.6 cells/field; mid-graft: 12.8 ± 3.2 cells/field) (Fig. 4E and F). These findings demonstrate that DMwVG significantly enhances the recruitment of circulating CD93^+^/CD31^+^ endothelial cells, thereby driving efficient fallout endothelialization.

**Fig. 5:**
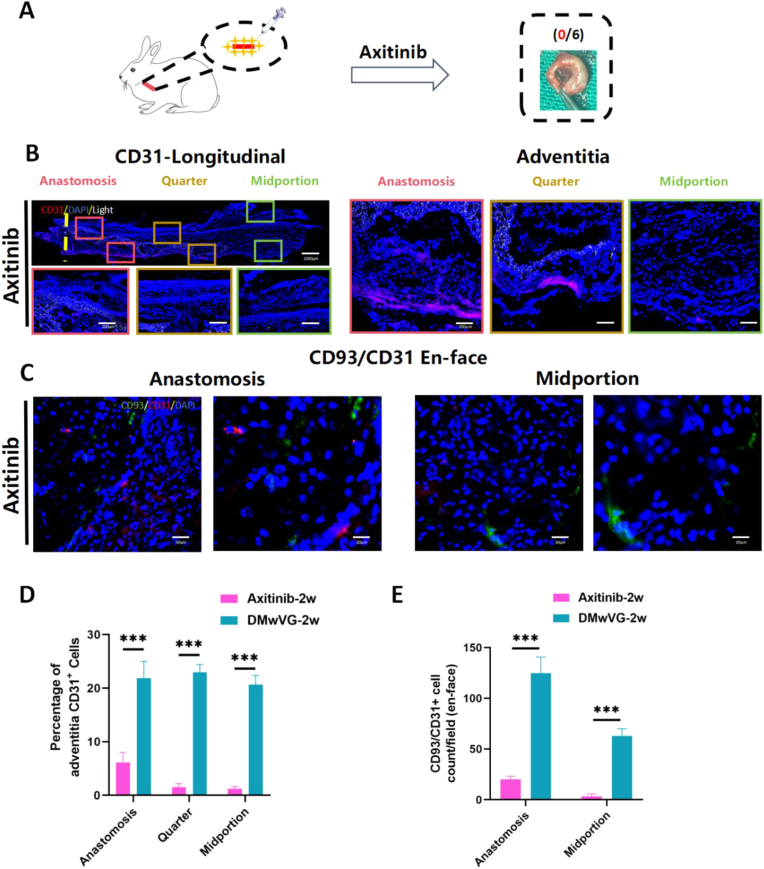
**Neo-adventitia angiogenesis enhanced fallout endothelialization.** (A) Schematic representation of axitinib injection. (B) CD31 immunofluorescence staining of longitudinal sections from various regions following axitinib administration at two weeks. (C) En-face analysis of CD93+/CD31+ cells at the anastomosis and mid-portion of neo-adventitia. (D) Quantification of adventitial and luminal CD31^+^ cells at different neoarterial sites (n = 5 independent samples). (E) Quantification of CD93+/CD31+ cells at the anastomosis and mid-portion of neo-lumen (n = 5 independent samples). For (D and E), significance was assessed using Student's t-test. **: P < 0.01, ***: P < 0.001.

To determine the role of vascularized neo-adventitia played in the fallout endothelialization observed in DMwVGs, six rabbits underwent DMwVGs implantation received perivascular injection of axitinib, which was a selective VEGFR inhibitor. All grafts were explanted at 2 weeks post-implantation for analysis. Remarkably, all six grafts in the axitinib group were completely coagulated at 2 weeks (Fig. 5A). Immunofluorescence staining of longitudinal sections for CD31 revealed significant differences in adventitial and luminal remodeling compared to the DMwVGs. In the axitinib group, both adventitia angiogenesis and luminal endothelial coverage were severely suppressed. En-face immunostaining was conducted to quantify the recruitment of circulating endothelial cells. The axitinib group exhibited a significantly lower density of CD93^+^/CD31^+^ endothelial cells on the luminal surface compared to the DMwVGs group (Fig. 5C and D). These findings suggest that adventitia angiogenesis plays a promotional role in early endothelialization. The inhibition of VEGFR significantly impeded early endothelialization, resulting in decreased graft patency.

To evaluate the remodeling process in lumen, the combination of histological and immunohistochemical techniques was used to characterize the intima remodeling. H&E staining analysis revealed rapid intima remodeling of DMwVGs. At two weeks post-implantation, a layer of compactly aligned cells, resembling endothelial cells, completely covered the luminal surface. A neotissue layer measuring 265.9 ± 48.0 μm in thickness was observed within the lumen (Fig. S5A). Throughout the period from 4 to 12 weeks, the neointima consistently produced concentrically aligned, wave-shaped ECM, gradually forming layered structures that resembled natural vessels. By 12 weeks, a more mature remodeling process was evident, characterized by peripheral capillaries exhibiting a radial distribution pattern. The lumen structure was well-defined, displaying typical elastic folds, while the intimal thickness stabilized at 290.5 ± 28.5 μm (Fig. 3D, Fig. S5A). The remodeled graft wall exhibited a trilayered structure similar to those of native arteries, maintaining an even wall thickness and becoming increasingly compact from 2 to 12 weeks. Despite the presence of minichannel constructs and adventitial encapsulation, no excessive scar hyperplasia was observed (Fig. S5A). Under polarized light, PGS residues exhibited a non-birefringent, darker appearance, whereas PCL fragments showed characteristic bright birefringence. Both PGS and PCL could still be identified at 2 weeks post-implantation (Fig. S5A). Quantification showed that PGS residues (23.5 ± 4.2% of the original area) were largely present at 2 weeks and completely degraded by 4 weeks (Fig. S5E). The minichannel design reduces the total polymer mass compared to a solid wall of the same outer diameter, which may facilitate fast tissue integration. By week 12, PCL residual area was minimal (3.6 ± 0.3 × 10^3^ μm^2^ per field) (Fig. S5D). This controlled degradation timeline effectively guided and accommodated the tissue ingrowth without mechanical compromise. To further understand the degradation behavior of the PGS/PCL blend scaffold, we characterized the polymer changes under *in vitro* enzymatic conditions using GPC, FTIR and SEM. GPC analysis revealed a progressive reduction in molecular weight, with Mw decreasing by 74.9% over 4 weeks, while FTIR spectroscopy confirmed ester bond cleavage as evidenced by weakening of the C–O–C peak (∼1245 cm^−1^) and concomitant strengthening of the C=O peak (∼1720 cm^−1^) (Fig. S9B–D). These chemical changes, together with the surface erosion observed by SEM, corroborate that the PGS/PCL scaffold undergoes hydrolytic degradation in a controlled manner. Elastic Van Gieson (EVG) staining, Masson's trichrome staining (MTS), and immunohistochemical staining were employed to characterize the elastin and collagen components. The deposition and maturation of functional ECM components followed a clear timeline. MTS analysis revealed the arrangement of collagen fibers, which exhibited an orderly circular pattern at 4 weeks post-implantation. By 12 weeks, the collagen fibers became densely arranged in an interconnecting lamellar network (Fig. S6A). EVG staining demonstrated that elastic fibers began to deposit at 4 weeks, subsequently forming concentric circular layers of elastic fiber bundles by 12 weeks (Fig. 4C). Immunofluorescence staining revealed that α-SMA positive cells were dispersed in the neointima and adventitia layers of the neoartery at 2 weeks. Over time, they gradually increased in number and aligned circumferentially. By 12 weeks, these cells were compactly arranged in the neointima of the neoartery (Fig. S6A and C), resembling the pattern observed in native arteries. Immunostaining confirmed that both collagen type I and type III became thicker and denser throughout the vascular wall by 12 weeks (Fig. S6B), achieving collagen content levels comparable to those of native vessels (Fig. S5F and G). Mechanical testing revealed that although the burst pressure and compliance at 12 weeks were slightly lower than native levels, the ultimate tensile strength of the regenerated vessels matched that of native arteries, thereby confirming the acquisition of physiologically relevant mechanical properties (Fig. S6D–F). In stark contrast, the high rate of thrombosis adversely affected neointimal regeneration in the DES group, resulting in a scarcity of functional ECM (Fig. S6A).

### 2.5 Neo-artery acquired similar structure and myography with native artery following long-term implantation

**Fig. 6:**
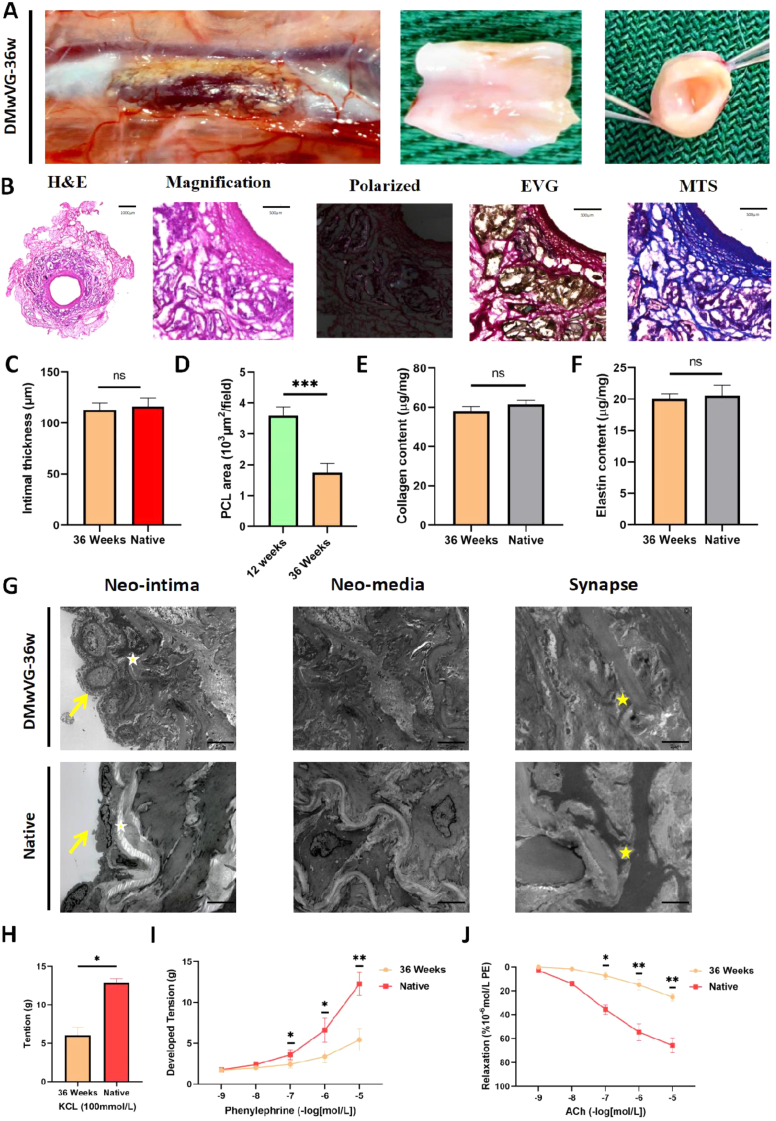
**Assessment of the neoartery at 36 weeks post-implantation.** (A) Gross section of neoarteries 36 weeks post-implantation. (B) Histochemical staining and polarized H&E staining of transverse sections at the midportion of neoarteries 36 weeks post-implantation. (C) Quantitative analysis of intimal thickness in neoarteries at 36 weeks (n = 6 in each group), with the native vessel serving as a control. (D) Quantitative analysis of PCL area in neoarteries at 36 weeks (n = 5 independent samples). (E-F) Collagen and elastin content in neoarteries at 36 weeks (n = 6 in each group). (G) TEM morphology of neoartery structure and native artery as control. In the neo-intima, the yellow arrowhead indicates ECs and the white star marks the basement membrane. In synapses, the yellow arrowheads indicate synaptic structures. (H-J) Examination of vasoreactivity in neoarteries using potassium chloride (H), phenylephrine (I), and acetylcholine (J) with n = 3 independent samples. For (C-F and H-J), significance was determined by Student's t-test. ns: P > 0.05; *: P < 0.05; **: P < 0.01; ***: P < 0.001.

A systematic analysis of DMwVGs 36 weeks after implantation was conducted due to the slow degradation rate of PCL. All implanted grafts (n = 6) remained patent, without evidence of aneurysm formation or intimal hyperplasia. Macroscopically, the residual PCL area progressively decreased, while the neoarteries remained well-integrated and exhibited abundant capillary ingrowth. The lumen was free of thrombus with the vessel exhibiting native-like elasticity and morphology (Fig. 6A). Histological analysis confirmed a trilayered structure similar to those of native arteries (Fig. 6B), with medial and intimal thicknesses comparable to native arteries (36 weeks: 113.0 ± 6.6 μm; Native: 116.0 ± 8.7 μm) (Fig. 6C). The minichannel remnants were confined to the neoartery adventitia, and the hollow structures were largely replaced by perivascular tissue. Polarized imaging revealed scattered fragments of PCL, and quantitative analysis confirmed progressive degradation (residual area: 1.7 ± 0.3 × 10^3^ μm^2^/field at 36 weeks vs. 3.6 ± 0.3 × 10^3^ μm^2^/field at 12 weeks) (Fig. 6D).

ECM remodeling in neoarteries exhibited a native-like organization, characterized by aligned type I and III collagen fibers, as well as elastic fibers within the medial layer. Masson's trichrome staining confirmed the presence of organized collagen fibers (stained blue) and smooth muscle bundles (stained red) in the neointima, while EVG staining revealed well-aligned black-stained elastic fibers (Fig. 6B). Quantitative analysis indicated no significant differences in elastin or collagen content compared with native arteries (Fig. 6E and F). Immunofluorescence analysis revealed a continuous monolayer of CD31^+^ cells lining the entire luminal surface of the neoarteries, with endothelial cell density comparable to that of native arteries. The density of adventitial capillaries continued to increase (159.6 ± 7.1 per field at 36 weeks vs. 126.4 ± 16.8 at 12 weeks) (Fig. S8A and C). This finding confirmed the presence of a confluent endothelial layer and underscored robust adventitia angiogenesis. Additionally, immunofluorescence staining for α-smooth muscle actin (α-SMA) demonstrated a uniform distribution of α-SMA^+^ cells, with no significant difference in coverage area compared with native arteries (Fig. S8B). Mechanical testing revealed that the burst pressure at 36 weeks matched that of native arteries, thereby confirming the acquisition of physiologically relevant mechanical properties (Fig. S8D–F).

Ultrastructural analysis using transmission electron microscopy (TEM) revealed a continuous internal elastic lamina, along with an interwoven network of elastic fibers, collagen bundles, and smooth muscle cells. Notably, synaptic connections between smooth muscle cells and nerve axons were identified by TEM, with distinct synaptic vesicles observed in presynaptic terminals, indicating reinnervation. The native artery exhibits a more flattened and mature endothelial morphology with a continuous luminal lining and mature synaptic structures, as was characterized by abundant synaptic vesicles and clearly defined cleft-like junctions (Fig. 6G). Functional assessments confirmed both contractile and vasodilatory capacities; however, the responses to KCl (6.0 ± 1.1 g tension) and phenylephrine (5.4 ± 1.3 g) were lower than those of native levels (12.8 ± 0.6 g and 12.3 ± 1.4 g, respectively). Endothelium-dependent vasodilation in response to acetylcholine was measured at 25.3 ± 3.0%, which was significantly lower than that of native arteries (65.7 ± 6.1%), suggesting ongoing functional maturation (Fig. 6H–J). In summary, DMwVGs facilitated stable structural regeneration of multilayered arterial constructs over 36 weeks. The neoarteries achieved excellent integration, biocompatibility, and progressive functional recovery, underscoring their long-term potential as small-diameter vascular grafts.

### 2.6 CXADR-mediated signaling enhanced angiogenesis of endothelial cells in neo-adventitia

**Fig. 7:**
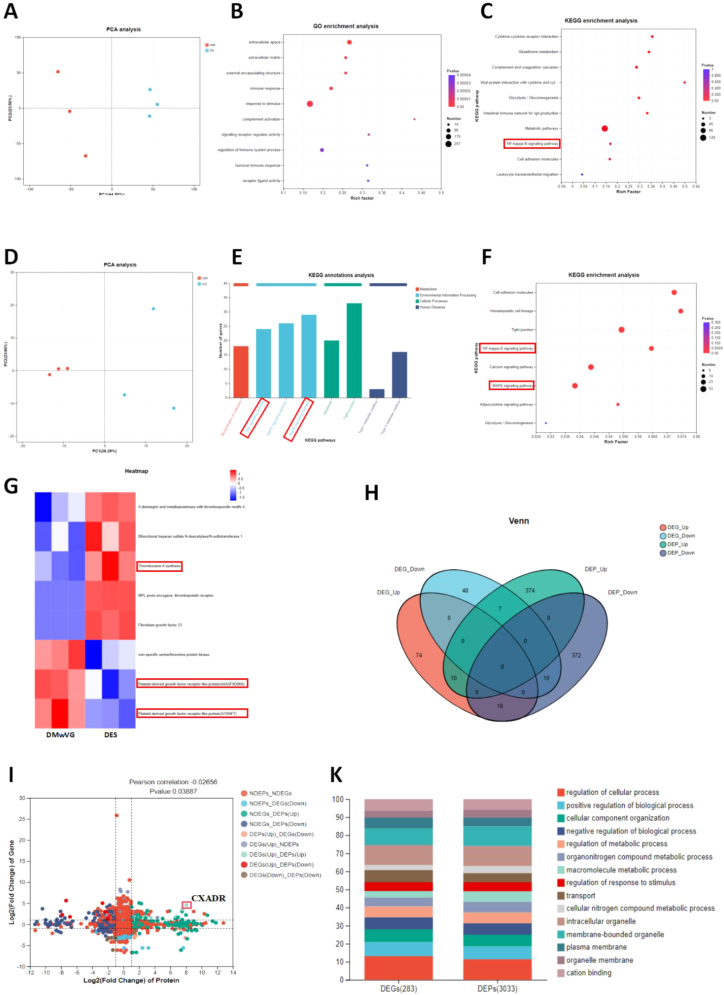
**The combined analysis results of proteomics and transcriptomics.** (A) Principal Component Analysis (PCA) of proteomics. (B and C) Gene Ontology (GO) enrichment and Kyoto Encyclopedia of Genes and Genomes (KEGG) enrichment analyses of proteomics. (D) PCA analysis of transcriptomics. (E and F) GO and KEGG enrichment analyses of transcriptomics. (G) A heatmap illustrating the cluster analysis of differentially expressed proteins.(H) Joint analysis of proteomics and transcriptomics utilizing Venn plots. (I) The nine-quadrant graph from the joint analysis confirms CXADR as a highly expressed target in both omics. (J) A stacked bar chart depicting the GO analysis for the joint study.

**Fig. 8:**
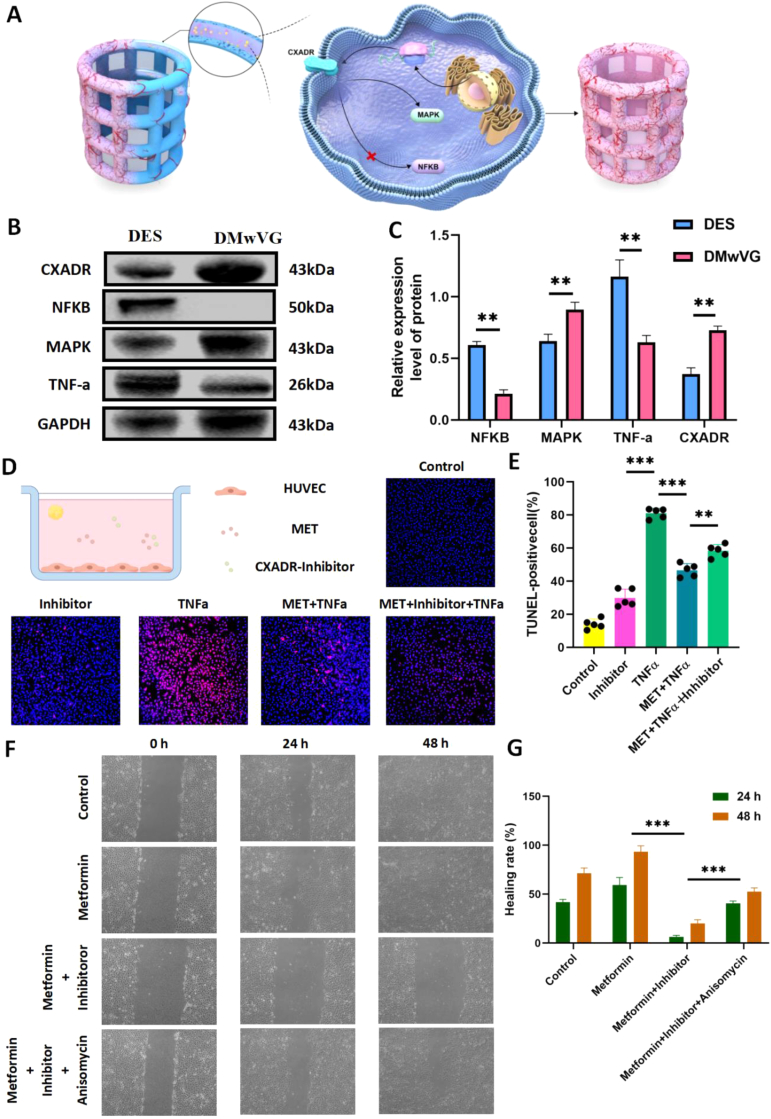
***In vitro* validation of CXADR-mediated signaling.** (A) Schematic representation of CXADR-mediated signaling pathways. (B and C) Western blot analyses for CXADR, NF-κB, MAPK, and TNF-α in both the DES and DMwVGs groups at the two-week mark (n = 3 independent samples). (D and E) Representative images from terminal deoxynucleotidyl transferase-mediated nick-end labeling (TUNEL) of HUVECs induced by TNF-α (TUNEL, red; DAPI, blue) (n = 5 independent samples). (F) Endothelial cell scratch assay of four groups: control group, metformin group, metformin + inhibitor (inhibition) group and metformin + inhibitor + anisomycin (rescue) group. (G) Quantification of healing rate in endothelial cell scratch assay (n = 5 independent samples). The plotted data are expressed as means ± SD. For (C), significance was assessed using Student's t-test. For (E and G), statistical significance was determined using one-way ANOVA followed by Tukey's post hoc analysis. **: P < 0.01, ***: P < 0.001.

To systematically investigate the molecular basis of adventitia angiogenesis observed in DMwVGs, we conducted integrated proteomic and transcriptomic analyses on the adventitia collected two weeks after implantation from DES and DMwVGs groups. Utilizing 4D label-free LC–MS/MS, we quantified 6953 of the 6954 detected proteins. Proteomic analysis revealed 918 differentially expressed proteins, with 464 upregulated and 454 downregulated. Principal component analysis confirmed the high quality of the data and revealed clear separation between the groups (Fig. 7A–D). Gene Ontology (GO) enrichment analysis highlighted biological processes related to ECM organization, immune regulation, and signal transduction (Fig. 7B–E). Notably, heatmap analysis revealed that DMwVGs significantly downregulated proteins associated with inflammatory responses and excessive fibrosis, while concurrently upregulating pro-angiogenic factors (Fig. 7G). Parallel transcriptomic analysis revealed 672 differentially expressed genes (DEGs), including 523 upregulated and 149 downregulated genes. Kyoto Encyclopedia of Genes and Genomes (KEGG) analysis demonstrated significant modulation of metabolic pathways, cell adhesion, and inflammatory signaling. Specifically, the MAPK pathway and TNF-α pathway were markedly promoted, whereas the NF-κB pathway was substantially inhibited (Fig. 7C–F). Integrated multi-omics analysis identified 45 molecules that were consistently differentially expressed across both omics layers (Fig. 7H). Nine-quadrant analysis further identified CXADR as a critical mediator, which was significantly upregulated at both the protein and mRNA levels (Fig. 7I). CXADR is known to facilitate intercellular adhesion and activate MAPK signaling via its ITAM motifs [29,30]. Consistent with this, Western blot analysis of adventitia corroborated the proteomic findings: compared with the DES group, DMwVGs upregulated CXADR expression by 3.1-fold, enhanced MAPK phosphorylation by 2.4-fold, and suppressed NF-κB activity by 62% (Fig. 8A and B). *In vitro*, while TNF-α potently induced endothelial cell apoptosis, the sustained-release metformin effectively counteracted this effect, restoring HUVEC viability (Fig. 8C and D). These results confirm that DMwVG promotes vascular remodeling through the CXADR-MAPK axis while inhibiting the NF-κB/TNFα pathway, thereby coordinating early angiogenic functions of endothelial cells in adventitia. To functionally validate the CXADR-MAPK axis, we performed *in vitro* scratch wound healing assays using HUVECs. Metformin treatment significantly accelerated wound closure compared with control, achieving a healing rate of 93.4 ± 6.1% at 48 h versus 71.4 ± 5.3% in the control group. However, co-treatment with a CXADR inhibitor (CXADR-blocking antibody) markedly suppressed this pro-migratory effect, reducing the healing rate to 20.2 ± 3.7% (P < 0.001 versus metformin alone). Notably, supplementation with anisomycin (10 μM), a MAPK activator, rescued the migration defect, restoring the healing rate to 52.5 ± 3.9% (P < 0.001 versus CXADR inhibitor group) (Fig. 8F and G). These data indicate that metformin promotes endothelial cell migration through the CXADR-MAPK signaling cascade.

**Fig. 9:**
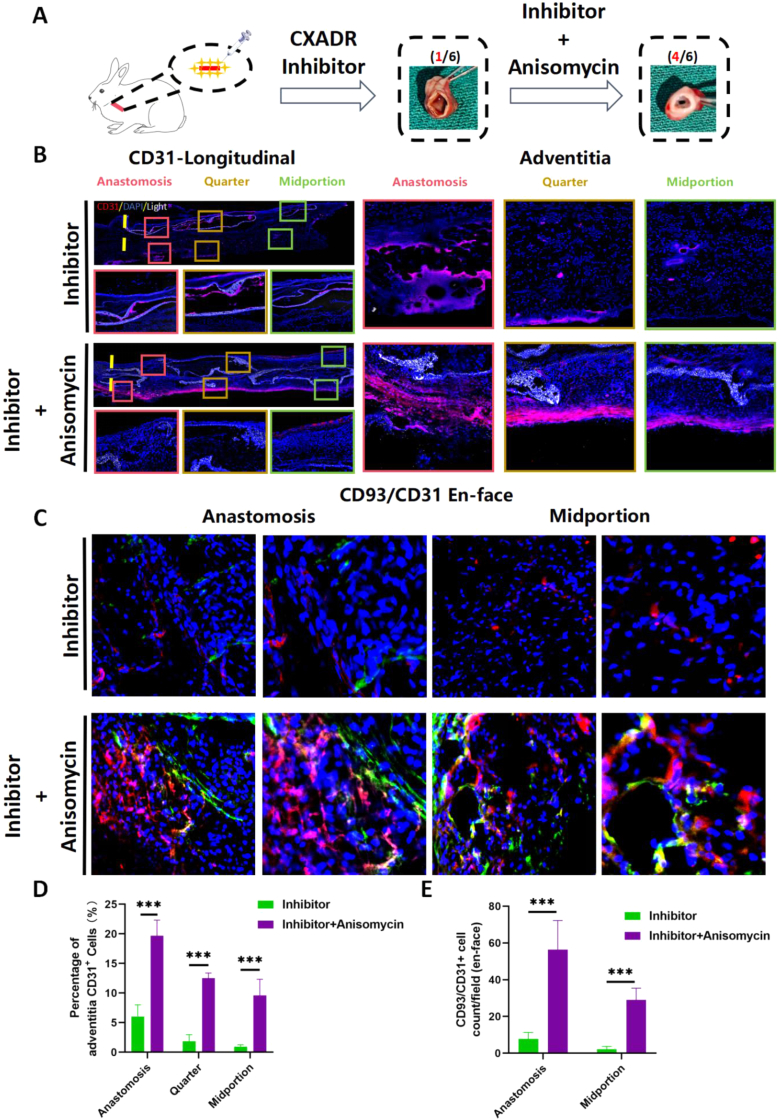
***In vivo* validation of the CXADR-MAPK signaling axis in DMwVG-mediated vascular remodeling.** (A) Schematic representation and patency rates of inhibitor and rescue groups. (B) Immunofluorescence staining of CD31 (red) in longitudinal sections of the graft at 2 weeks. (C) En-face analysis of CD93+/CD31+ cells at the anastomosis and mid-portion. (D) Quantification of adventitial and luminal CD31^+^ cells at different sites (n = 5 independent samples). (E) Quantification of CD93+/CD31+ cells at the anastomosis and mid-portion of two groups (n = 5 independent samples). For (D and E), significance was assessed using Student's t-test. **: P < 0.01, ***: P < 0.001.

We then examined the functional relevance of CXADR-MAPK signaling *in vivo*. A separate cohort of rabbits receiving DMwVGs was treated with perivascular injection of a CXADR inhibitor (n = 6) or a combination of CXADR inhibitor and anisomycin (n = 6) (Fig. 9A). At 2 weeks post-implantation, the patency rate in the CXADR inhibitor group dropped to 17% (1/6), whereas co-administration of anisomycin significantly rescued patency to 67% (4/6). Immunofluorescence analysis revealed that CXADR inhibition drastically reduced both CD31^+^ capillary density and luminal CD93/CD31^+^ cells coverage, while anisomycin partially restored these parameters (Fig. 9D-E). These results establish a causal role of CXADR-MAPK signaling in metformin-mediated adventitial neovascularization and fallout endothelialization, and corroborate the multi-omics findings that this pathway is a critical mediator of the therapeutic effects of DMwVGs. Collectively, these results delineate a central mechanism: DMwVGs promote rapid adventitial vascularization via activation of the CXADR-MAPK pathway while suppressing the NF-κB/TNF-α axis.

## 3 Discussion

Unlike conventional vascular grafts that primarily focus on surface antithrombotic modifications, this study introduced a synergistic strategy by actively promoting adventitia regeneration. To address the limited efficiency of drug loading, we developed 3D minichannels for perivascular drug administration, which enabled sustained release of metformin within the perivascular niche. As compared with DES, DMwVGs increased drug loading capacity by 4.4 times, significantly accelerated adventitia angiogenesis and enhanced the recruitment of lymphatic endothelial cells, thereby promoting fallout endothelialization and ensuring long-term patency. To the best of our knowledge, this study represents the first demonstration of vascular grafts designed to specifically target adventitia regeneration, providing critical validation for the concept of perivascular drug administration.

The formation of a complete endothelial layer relies on three fundamental elements: an adequate supply of endothelial (progenitor) cells, precise signals for cell homing and adhesion, and a porous surface conducive to cell survival and proliferation [31,32]. Current strategies to promote endothelialization primarily focus on functionalizing the luminal surface of vascular grafts, including coating with bioactive molecules such as heparin or RGD peptides to inhibit thrombosis and enhance endothelial adhesion; incorporating growth factors such as VEGF to recruit circulating EPCs; or pre-seeding endothelial cells within the lumen [[33], [34], [35]]. Although these approaches demonstrated some success, they are constrained by limitations such as low drug-loading capacity, uncontrolled release kinetics, the susceptibility of active molecules to deactivation under blood dilution, and the operational complexity associated with cell seeding. Due to the slow migration rate of native endothelium from anastomotic sites in humans, fallout endothelialization, which relies on the cooperative action of surrounding tissues and circulating cells, may be more efficient for vascular grafts with clinically relevant lengths. Pennel et al. showed that perivascular tissue can provide smooth muscle-like cells, immune cells, and endothelial cells to artificial blood vessels, thereby promoting vascular remodeling [36]. Clowes et al. demonstrated that increasing the pore size of vascular grafts enhances tissue ingrowth and achieves complete re-endothelialization of ePTFE grafts in a baboon model [37]. Building on these insights, this study proposes a strategic shift: redirecting the regulatory focus from the lumen to the adventitia regeneration. We believed that the adventitia and perivascular tissue constitute an active regenerative niche, as is rich in precursor cells and capable of modulating luminal remodeling. To leverage this, we designed a three-dimensional minichannel system wrapped around the PCL electrospinning graft. These minichannels functioned as high-capacity, controlled-release drug reservoirs that facilitated the sustained release of metformin, thereby promoting robust adventitia ingrowth and indirectly enhancing luminal endothelialization. Recent reviews highlighted 4D printing as a transformative approach for creating adaptive biomedical devices, including stents, drug delivery systems, and tissue scaffolds [[38], [39], [40]]. Aligned with this paradigm, our metformin-loaded minichannels achieved a different but complementary form of perivascular drug administration that actively regenerated the adventitial niche over time to promote vascular graft remodeling.

Metformin was selected as the primary therapeutic agent due to its pleiotropic biological effects, including anti-inflammatory, antioxidant, pro-angiogenic, and endothelial-protective properties. Hu et al. demonstrated that metformin enhances microvascular dysfunction by inhibiting mitochondrial fission and promoting chronic diabetic wound healing [41]. Our previous research claimed that a metformin-loaded 3D-printed stent facilitates the reconstruction of vein grafts following perivascular tissue deprivation, preventing early thrombosis and subsequent intimal hyperplasia [23]. In alignment with these findings, the sustained release of metformin from the minichannels in this study enhanced the angiogenic properties (Fig. 2A–H), which significantly stimulated adventitia angiogenesis and enhanced the recruitment of lymphatic endothelial cells (Fig. 3D–J). Mechanistically, the sustained release of metformin promotes a pro-angiogenic and anti-inflammatory adventitial regenerative niche by inhibiting the NF-κB/TNF-α pathway, which reduces inflammation and fibrosis, while concurrently upregulating the CXADR-MAPK pathway to enhance cell adhesion and proliferation. Recent research has shown that CXADR is capable of triggering and collaborating with various signaling pathways to initiate diverse downstream effects. Farmer et al. conducted studies in epithelial cells that demonstrated CXADR's interaction with integrin receptors, inducing the activation of the p44/42 MAPK pathway through CXADR, followed by the activation and localization of β1 and β3 integrins at cell-cell junctions [42,43]. In consistence with this, multi-omics analysis corroborated that this niche is characterized by upregulated pro-angiogenic factors and downregulated pro-fibrotic proteins, which attracted host precursor cells, promoted capillary formation, and accelerated tissue integration. Furthermore, the controlled-release design is crucial to this strategy. In contrast to the burst release observed in the DES group, which exhibited an 80% release within 72 h, the minichannel-collagen strategy achieved a steady release over several weeks (Fig. S1J). This durable and stable local delivery maintained an effective therapeutic concentration in the adventitial region, effectively suppressed acute inflammation early while continuously offered tissue regeneration signals. The significantly lower early patency rate (33%) and degree of endothelialization in the DES group, compared to the DMwVG group (83%) strongly underscored the necessity of localized, controlled perivascular drug administration for artificial vascular remodeling (Fig. 3B).

The efficient endothelialization observed in DMwVGs aligned with the paradigm of fallout endothelialization, which relied on circulating endothelial cells rather than transanastomotic endothelial cell growth. Theoretically, circulating blood is rich in endothelial progenitor cells (EPCs), which are a group of early-stage cells capable of differentiating into endothelial cells [[44], [45], [46], [47]]. In 1997, Asahara et al. first isolated EPCs from human peripheral blood, demonstrating their contribution to vascular repair and regeneration in adults [48]. Bianconi et al. enhanced endothelial repair in patients with cardiovascular disease using EPC-capturing stents [49]. Our previous studies have shown that CD93^+^/CD31^+^ endothelial cells in circulating blood were crucial for the early endothelialization of rabbit carotid artery grafts, primarily due to their high migratory and proliferative potential [17,28]. In this study, unlike the traditional model that relies on transanastomotic endothelial cell growth, the DMwVGs group exhibited clusters of CD93^+^ endothelial cells in the quarter and mid-graft region, which aligned with the principles of fallout endothelialization (Fig. 4E–F). However, it should be noted that the distribution of endothelial cells was not uniform, with more ECs recruited near the anastomotic sites and slower endothelialization in the midportion of the grafts (Fig. 3B–D). The key to enabling this efficient fallout endothelialization lay in our strategy of promoting rapid adventitia angiogenesis. Clinically, perivascular drug administration is frequently employed with antiproliferative agents to inhibit excessive smooth muscle cell proliferation following vascular injury, thereby preventing intimal hyperplasia [50,51]. In contrast, our perivascular drug-loaded minichannels aim to promote rapid transmural growth of vascularized neo-adventitia, thereby driving efficient endothelialization. A stark contrast was observed between the two groups regarding adventitia angiogenesis. CD31^+^ cell coverage in the DMwVG group increased progressively across the anastomotic, quarter, and mid-graft regions (Fig. 3D–G). This spatially homogeneous neovascularization indicated that DMwVGs promoted robust recruitment of perivascular tissue. In stark contrast, the DES group exhibited sparse peripheral capillary formation and was encapsulated by a thin fibrous layer, with CD31^+^ cell densities in the neo-adventitia remaining significantly lower. These findings highlighted the superior capacity of DMwVGs to induce rapid adventitia angiogenesis, which was essential for subsequent tissue integration and graft remodeling. Furthermore, immunofluorescence staining revealed abundant lymphatic endothelial cells within the DMwVGs adventitia at 2 weeks, a feature absent in the DES group (Fig. 3I and J). This finding suggested that the regenerated neo-adventitia functioned not merely as a passive scaffold but as an active signaling and cellular reservoir that promoted luminal endothelialization. Our previous research suggested that the crosstalk between lymphatic endothelial cells in the neo-adventitia and circulating endothelial cells plays a crucial role in early endothelialization [17]. In line with this, this study provided additional supporting evidences: (1) During early implantation, CD31^+^ cells were distributed around adventitial minichannels, with circulating CD93^+^ cells recruited to the luminal side; (2) The facilitated endothelialization resulted in early, multicentric, and rapid endothelial coverage at the quarter and mid-portion of the graft, demonstrating significantly higher efficiency compared to traditional anastomosis-dependent migration (Fig. 4B–D); (3) Inhibition of VEGFR completely abolished luminal endothelialization, resulting in thrombosis in all grafts (Fig. 5B–E). Ideally, the luminal surface should promote the specific adhesion and growth of EPCs while inhibiting platelet adhesion and thrombus formation. Additionally, Jason et al. demonstrated that excessively thick vascular walls may hinder the remodeling of artificial blood vessels [52]. The combination of reduced porosity and increased stiffness may impair the transmural communication between the adventitia and the lumen, thereby limiting the signals from the perivascular niche [17]. Consistent with this, the 125 μm electrospun layer prone to thrombosis, while the 75 μm electrospun layer provided sufficient hemocompatibility and facilitated the migration of endothelial cells, which was essential for maintaining the permeability of the graft walls and ensuring signaling communication between the neo-adventitia and circulating cells. Collectively, these findings demonstrated that DMwVGs reprogramed the adventitia into a pro-regenerative niche, facilitating fallout endothelialization through localized perivascular minichannels, thereby providing alternative direction for small-diameter vascular graft design.

Nevertheless, the vasodilatory response observed at 36 weeks likely does not represent the final mature state, as our study has already demonstrated that the neoarteries achieved basal contractile function (responses to KCl and phenylephrine) and regained partial endothelium-dependent relaxation, indicating their potential for continued maturation toward physiological function. Despite the near-complete endothelial coverage and organized trilayered structure observed at 36 weeks, the vasodilatory response of the regenerated neoartery to acetylcholine remained significantly lower (25.3%) than that of the native carotid artery (65.7%), indicating ongoing functional maturation. Several interconnected mechanisms may limit full vasoreactivity at this time point. First, immature neural–endothelial coupling: although synaptic structures were identified by transmission electron microscopy (Fig. 6G), the functional communication between autonomic nerve endings and the endothelium may not yet be fully operational. Neural reinnervation is a protracted process; even when morphological synapses are established, neurotransmitter release and receptor responsiveness often require additional time to reach physiological levels [53]. Second, transitional endothelial phenotype: the regenerated endothelium may still reside in an intermediate state, between an immature and a fully quiescent-activated phenotype [54]. Accumulating evidence indicates that tissue-engineered blood vessels typically demand extended remodeling periods to attain complete vasodilatory function, especially when an endogenous source of endothelial progenitor cells is lacking. Third, suboptimal mechanical microenvironment: although the compliance and diameter of the neoartery closely approximate those of native vessels (Fig. S8E–F), the wall stiffness and luminal surface topography may not yet perfectly recreate the native mechanical milieu. This imperfection could impair the signal integration capacity of mechanosensitive molecules such as PIEZO1 and VE-cadherin. To improve long-term vasoreactivity in future iterations, we propose several design modifications: (i) co-delivery of pro-contractile factors such as transforming growth factor-β1 (TGF-β1) or platelet-derived growth factor (PDGF) from the perivascular minichannels to promote smooth muscle maturation; (ii) incorporation of aligned electrospun fibers or micropatterned surfaces in the inner layer to guide oriented smooth muscle cell organization; and (iii) staged release of vasoactive peptides (e.g., C-type natriuretic peptide) after the initial endothelialization phase to enhance NO signaling [55]. These strategies could help achieve more complete functional restoration of tissue-engineered vascular grafts.

Despite the promising results, it is essential to acknowledge the limitations of this study. Although small animal models are valuable for the early screening of antithrombotic capacity and the long-term biological responses of tissue-engineered blood vessels, large animal models are crucial for preclinical testing of vascular grafts, as they more closely resemble human anatomy and hematology. Among them, sheep and pigs are often considered suitable models (excluding non-human primates) due to their similarities to humans in thrombosis, endothelialization, and lipoprotein metabolism [56,57]. Therefore, future studies should concentrate on validation in large-animal models and the development of feedback-controlled release systems to enhance therapeutic precision and expedite clinical translation. Moreover, as the thickness of the PCL electrospun layer increases to meet higher arterial burst pressure requirements, further optimization of the material structure will be necessary in preclinical applications to balance the trade-off between thickness and mechanical strength.

## 4 Experimental section

### 4.1 Fabrication of sacrificial caramel templating

Caramel-based templates were 3D printed via fused deposition modeling. To produce 3D-printed caramel, sucrose (Macklin, China), glucose (Macklin, China), fructose (Macklin, China), and dextran (Macklin, China) were weighed at a mass ratio of 50 g:15 g:10 g:10 g, mixed with 40 mL of distilled water, and stirred at a constant temperature of 130°C until completely dissolved. After the sugar particles were fully dissolved, the mixture was placed in a 130°C constant-temperature oven for 12 h for caramel preheating while preventing excessive oxidation in air. The caramel ink was printed into a double-layer structure at 100-110°C. The printing temperature was determined based on the extrusion flow state of the caramel ink, as its fluidity is highly sensitive to temperature variations within this range. All parameters for the caramel-based template are as follows: nozzle diameter of 0.2 mm, layer height of 0.2 mm, intersection angle between adjacent layers at 90°, material length, width, and height set to 3 cm, 1.2 cm, and 0.09 cm respectively, infill rate of 20%, extrusion speed of 5 mm/s, and air pressure at 0.5 MPa. After cooling in air, the caramel tubular scaffold can be easily removed from the printing platform, then bent into a tubular shape in a 40°C environment, with the edges welded using a heating rod.

### 4.2 Fabrication of 3D minichannel-wrapped vascular grafts (MwVGs)

Based on these 3D-printed caramel templates, we weighed polycaprolactone (PCL: Sigma, USA) and PGS (synthesized internally as previously reported) at mass ratios of 8:2, and dissolved them in hexafluoroisopropanol (3%, w/v). A magnetic stirrer was added and stirred at a constant temperature of 60°C at 500 rpm until the polymers were completely dissolved. Sodium chloride (Macklin, China) with a particle size of 30–38 μm was then added to the above solution as a porogen at a mass ratio of 1:2 to the polymer. After thorough mixing, the mixture was placed on ice. The caramel-based templates were immersed in the mixture solution and air-dried. The PCL:PGS coating materials with mass ratios of 8:2 were repeatedly prepared in 3-times, 6-times, and 9-times configurations respectively. After complete air-drying, they were immersed in distilled water with water replacement every 2 h until the caramel was completely dissolved. For three independent fabrication batches (n = 20 grafts per batch), the failure rate (grafts with >20% of channels occluded or collapsed) was 6.3% across batches. Due to the caramel occupying the complete minichannel structure, the channel continuity rate (percentage of minichannels with uninterrupted flow as confirmed by dye perfusion) was 100%. As previously described, the PCL sheath was fabricated using electrospinning technology. Briefly, PCL was dissolved in trifluoroethanol at a concentration of 14% (w/v). Using a syringe pump, the PCL solution was supplied through a 22-gauge stainless steel needle at a rate of 1.5 mL/h for 3 min while applying a high voltage (26 kV). Prepared a cocktail drug solution containing 20 mmol/L MET dissolved in 8% bovine collagen solution. Slowly injected the solution into the microchannel of dry DMwVGs using a 1 mL syringe until the microchannels were completely perfused with the cocktail/collagen solution. After overnight storage at −80°C, lyophilized the DMwVGs in a vacuum freeze-dryer at −0.1 MPa and −80°C for 24 h. For the MwVGs group, prepared collagen scaffolds loaded with PBS.

### 4.3 Surface modification strategies for PCL electrospinning (ES)

A heparin-binding approach was utilized to apply DTβ4 onto the PCL electrospun scaffold (ES), following previously established protocols [16]. The scaffold was treated with a 0.1 M ethylenediamine solution while gently shaking for 60 min, after which it underwent three washes with distilled water. Next, the ES was placed in a MES solution (pH 5.6) that contained 1-ethyl-3-(3-dimethylaminopropyl) carbodiimide hydrochloride (0.1 M), N-hydroxysuccinimide (0.1 M), and heparin (10 mg/mL). The heparinization was carried out at 4°C over a 12-h period. Subsequently, the ES was cleaned by rinsing it three times with distilled water, resulting in a heparinized scaffold. The ES was then submerged in a DTβ4 solution (1 mg/mL) and allowed to incubate in an ice bath for 2 h. For the drug-eluting scaffold (DES), after three washes with distilled water to eliminate surplus impurities, electrospinning was executed on the exterior using a 20 mmol/L metformin-collagen mixture. In conclusion, both the ES and DES underwent freeze-drying under vacuum, were sterilized with ethylene oxide, and were stored at −20°C.

### 4.4 Fourier transform infrared spectroscopy measures absorption peaks

To determine whether the drugs were successfully loaded onto the 3D MwVGs, we conducted component analysis using Fourier transform infrared spectroscopy (FTIR) on both pure collagen-loaded scaffolds and metformin-loaded scaffolds. The scaffolds from both groups were fixed onto optical metal substrates, then scanned at a resolution of 4 cm−1 using a Thermo Nicolet IS 5 spectrometer, with spectra collected by OMNIC 7.3 software.

### 4.5 Mechanical characterization

The tensile and compressive strengths of the grafts were evaluated with a force testing system (BOSE, ElectroForce 3200 Series II, USA). For this assessment, MwVG segments were sectioned into lengths of 5 mm (n = 5). A uniaxial tensile load was applied to the segments at a speed of 5 mm/min, beginning with a force of 0.1 N until failure occurred. Each graft (n = 5) underwent compression to achieve 50% strain for measuring compressive force. The burst pressure of PCL electrospun layers and neoarteries, in addition to the compliance of the neoarteries, were assessed utilizing a perfusion system (MFCS-EZ microfluidic flow control system, Fluigent, France). The porosity was determined by mercury intrusion porosimetry (AutoPore IV 9500, Micromeritics). Samples were vacuum-dried at 40°C for 24 h, then loaded into a calibrated penetrometer and subjected to low-pressure (0.5–30 psia) and high-pressure (up to 60,000 psia) analyses. Pore size distribution was calculated using the Washburn equation with a mercury contact angle of 140° and a surface tension of 480 dyn/cm. Data are presented as mean ± SD from n = 3 independent samples per group.

### 4.6 *In vitro* and *in vivo* metformin release and tissue accumulation assays

We measured the *in vitro* sustained-release efficiency of MET loaded on the transplant collagen through the following procedures: ES and DMwVGs (1 cm) were dynamically incubated in RPMI-1640 (1 mL, containing 1% BSA) at 37°C for 28 days, with culture medium collected and replaced at different time points. The metformin sustained-release concentration in the collected medium was detected using an enzyme-linked immunosorbent assay (ELISA) kit, with measurements repeated three times. ES and DMwVGs (1 cm) were dynamically incubated in PBS containing 0.1% BSA and 0.01% collagenase under orbital shaking at 60 rpm at 37°C. At designated time points, the medium was collected and replaced with fresh medium. The metformin concentration in the collected medium was quantified using LC-MS/MS, with measurements performed in triplicate. To quantify local drug accumulation, perivascular tissues (adventitia and adjacent tissue within 1 mm of the graft) were harvested from a separate cohort of rabbits (n = 3 per time point) at days 3, 6, 9, 12, and 15 post-implantation. Tissues were homogenized in PBS, followed by protein precipitation with acetonitrile containing 0.1% formic acid. After centrifugation, the supernatant was analyzed by LC-MS/MS using metformin-d6 as an internal standard. Metformin concentration was calculated as μg per gram of tissue wet weight (μg/g). All measurements were performed in triplicate.

### 4.7 *In vitro* release profile of Cy3-labeled metformin in matrigel

Cy3 fluorescently labeled metformin was loaded into minichannels to assess its sustained-release efficiency within Matrigel, thereby simulating an *in vivo* sustained-release environment. Grafts were embedded in Matrigel, and fluorescence labeling was observed at depths of 100 μm, 200 μm, 300 μm, 400 μm, and 500 μm using confocal laser scanning microscopy at 24 and 48 h, respectively. The fluorescence intensity at each site was quantified using ImageJ software.

### 4.8 *In vitro* evaluation of blood compatibility

For the evaluation of anticoagulation, three distinct types of scaffolds were created and then electrospun using solutions of PCL mixed with PBS, heparin, and DTβ4, respectively. Each scaffold was sectioned with a custom circular blade featuring an 8 mm radius. A volume of 1 mL of fresh anticoagulated whole blood was recalcified and incubated with each scaffold sample in an EP tube at 37°C for a duration of 10 min. After this incubation, the grafts embedded with blood clots underwent five washes with PBS to eliminate any unbound red blood cells. Subsequently, the samples were preserved in a 2.5% glutaraldehyde solution in PBS at 4°C for 6 h. After fixation, the samples were processed following the established protocol for SEM sample preparation and were then examined using a PhenomXL scanning electron microscope (SEM) produced by Phenom-World (Netherlands).

### 4.9 Cell cultured

HUVECs were obtained from the American Type Culture Collection (ATCC) cell bank (Shanghai, China). The cells were cultured in Dulbecco's modified Eagle's medium/high glucose (HyClone, Logan, UT, USA) supplemented with 10% fetal bovine serum (Gibco, Grand Island, NY, USA). When reaching 70% confluence, all cells were passaged, and the culture medium was replaced every 2 days. Experiments were performed when the cells were at passages 2-4.

### 4.10 *In vitro* HUVECs apoptosis and HUVEC tube formation assay

The procedure for cell culture followed the previously outlined methodology. HUVECs were cultivated in four distinct conditioned media and subsequently seeded into 96-well plates that had been pre-coated with Matrigel (BD Pharmingen, San Diego, CA) before being incubated at 37°C. After a period of 6 h, the formation of endothelial cell tubes was examined using an optical microscope. The capillary-like tubular structures observed in five different fields were counted and documented through scanning. The apoptosis of HUVECs was assessed via the terminal deoxynucleotidyl transferase-mediated dUTP nick end labeling (TUNEL) assay, utilizing a detection kit provided by 4A Biotech Co., Ltd. in China. In summary, post-treatment, HUVECs were incubated with TdT and fluorescein-labeled dUTP at 37°C for 45 min. This was followed by a co-incubation with 4′,6-diamidino-2-phenylindole (DAPI) for nuclear labeling. Images were obtained with a Nikon (Japan) FV1000 laser scanning confocal microscope.

### 4.11 *In vitro* scratch wound healing assay

HUVECs were cultured to 100% confluence in 6-well plates. A uniform scratch was created using a 200 μL pipette tip. Cells were washed with PBS to remove detached cells and then incubated with serum-free medium containing the designated treatments: control (vehicle), metformin (5 mM), metformin + CXADR inhibitor (CXADR-blocking antibody), or metformin + CXADR inhibitor + anisomycin (10 μM). Wound closure was monitored at 0, 24, and 48 h under an inverted microscope (Nikon, Japan). The wound area was measured using ImageJ software, and the healing rate was calculated as: (1 − [wound area at t]/[wound area at 0 h]) × 100%. Experiments were performed in triplicate.

### 4.12 Transwell migration assay of HUVECs

The migratory ability of HUVECs was assessed using the Transwell assay. Cells in the logarithmic growth phase were resuspended in culture medium (0, 2.5, 5 mmol/L) and adjusted to a concentration of 2 × 10^5^/L. A 100 μL cell suspension was added to the upper chamber of the Transwell, while 0.6 mL of complete medium containing three types of scaffolds—MwVGs, DES, and DMwVGs, all cut with a customized 8-mm radius circular blade—was added to the lower chamber. After 24 h of incubation, cells on the upper surface of the Transwell membrane were removed, and cells on the lower surface were stained with crystal violet, observed under a microscope, photographed, and counted.

### 4.13 Animal experiments

The experimental rabbits used in this study were sourced from the Animal Center of the Air Force Medical University in Xi'an, China. Approval for all animal-related procedures was granted by the Animal Experiment Ethics Committee of the same institution, ensuring adherence to animal welfare guidelines. A total of 35 male New Zealand white rabbits, aged 6 months and weighing between 3.5 and 4.5 kg, underwent interposition grafting of the left carotid artery using 15 mm vascular grafts, distributed as follows: 6 in the MwVG group, 6 in the DES group, and 18 in the DMwVG group. For the acute anticoagulation evaluation test conducted *in vivo*, 15 mm long arterial grafts were utilized for the carotid artery interposition; this involved 5 grafts from each of the MwVG, DES, and DMwVG groups, which were subsequently retrieved after 2 h. Following the surgical procedure, a systemic anticoagulation therapy lasting one week was administered as part of the routine postoperative care, while all subjects were maintained under a 12:12-h light-dark schedule. Sample sizes were determined based on a power analysis using preliminary patency data. To detect such a difference with α = 0.05 and β = 0.20, a minimum of 5 animals per group was required. For the DMwVG group, we enrolled 18 animals to allow for evaluation at three time points (2, 4, 12 weeks; n = 6 per time point) plus additional animals for mechanistic studies (axitinib, inhibitor injections). Control groups (MwVGs, DES) were evaluated primarily at the 2-week patency endpoint (n = 6 each) because they were expected to have low patency and were not followed long-term. The procedure was performed by operators who were unaware of the specific drug-eluting grafts utilized. For inhibitor studies, three separate cohorts were used: (1) axitinib group (200 μL containing 200 μg axitinib, n = 6); (2) CXADR inhibitor group (200 μL containing 200 μg CXADR-blocking antibody (Med.Bio.Labs), n = 6); and (3) rescue group (200 μL containing 200 μg CXADR inhibitor combined with 100 μg anisomycin, n = 6). All animals were euthanized at 2 weeks for analysis. This procedure was approved by the Animal Ethics Committee.

### 4.14 Ultrasound patency monitoring

Monitoring of blood flow and vessel integrity in the grafted vessels was conducted through high-frequency ultrasound imaging. The diameter and blood flow velocity of both the native contralateral carotid artery and the implanted vessel were evaluated on days 14, 30, and 90 post-implantation, employing the Vevo 2100 ultrasound imaging system (FUJIFILM VisualSonics, Toronto, Canada). Isoflurane (1-2%) was used to induce anesthesia in the animals, which was administered via a precision vaporizer and gas flow control device. Long-axis images of both the common carotid artery and the graft were captured throughout the cardiac cycle using B-mode ultrasound, as well as color Doppler and pulsed-wave Doppler modes. The dynamics of playback images were analyzed using Vevo 2100 software to assess graft patency, variations in lumen diameter, and blood flow velocity in and around the graft. At the 2-week postoperative ultrasound assessment, 15 out of 18 animals (83%) were patent. Among these 15 patent grafts, 3 belonged to the 2-week subgroup (the other 3 in this subgroup were non-patent and were excluded from further analysis), while all 6 animals in the 4-week subgroup and all 6 in the 12-week subgroup were patent at 2 weeks. At their scheduled endpoints, all 6 animals in the 4-week subgroup remained patent (6/6), and all 6 in the 12-week subgroup remained patent (6/6). Thus, the overall 2-week patency for DMwVGs was 15/18 (83%), and all 15 patent grafts maintained patency until their scheduled explant.

### 4.15 Biochemical evaluation

Tissues from neointimal arteries and native carotid arteries were collected for collagen and elastin quantitative analysis (n = 5 independent samples). Total elastin was extracted from each explant and quantified using the Fastin Elastin Assay kit (F2000; Biocolor Ltd, Carrickfergus, UK) following the manufacturer's protocol. The data were subsequently combined and adjusted based on the sample's wet weight to calculate the mass of insoluble elastin for each unit of tissue wet weight (μg/mg). To assess the collagen content, the overall collagen in each explant was measured utilizing the Sircol Collagen Assay kit. The collagen concentration in the combined supernatant was determined following the guidelines of the kit, and the amount of insoluble collagen per gram of wet tissue weight was computed using a standardized collagen curve.

### 4.16 Histology evaluation

The neointima was embedded using OCT (Sakura Finetek, USA) and then processed for cryosectioning to obtain sections with a thickness of 12 μm. Following this, the sections were soaked in hematoxylin for 10 min, after which they were treated with 70% ethanol and eosin for 5 min. Subsequently, dehydration was carried out using ethanol, and the samples were subjected to xylene for decolorization, which finalized the H&E staining procedure. Masson staining (G1340, Solarbio, Beijing, China) and EVG staining (ab150667, Abcam, MA, USA) were conducted according to the protocols provided by the manufacturers of the reagents. All histological images were examined using an upright fluorescence microscope (DM6000 B, Leica, Germany) under either bright field or polarized light.

### 4.17 Quantification of PGS/PCL residuals in grafts

The calculation of the residual polymer quantity followed established methodologies. At designated time intervals, polarized H&E images from various samples (n = 5) within each group were employed to assess the extent of polymer residue. Under polarized light, PCL residues displayed distinctive white birefringence, whereas PGS did not show any birefringence. Bright-field microscopy revealed that PGS residues appeared as lightly stained, porous formations. The measurement of polymer residues was conducted using ImageJ software (National Institutes of Health).

### 4.18 Immunofluorescence staining

The samples were fixed with ice-cold acetone, permeabilized with 3% hydrogen peroxide, and then blocked in serum not derived from the primary antibody source for 60 min. The following primary antibodies were used in the experiment: CD31 (1:300 dilution; Abcam, ab6994), CD31 (1:300 dilution; Novus, NB 600-562), α-SMA (1:300 dilution; Abcam, ab242395), Collagen I (1:300 dilution; GeneTex, GTX 26308), and Collagen III (1:300 dilution; GeneTex, GTX 26310). The samples were incubated with the primary antibodies at 4°C for 12 h. After washing with PBS, the samples were incubated with fluorescent secondary antibody (1:1000 dilution; Abbkine) at 37°C for 1 h. The nuclei were stained with 4′,6-diamidino-2-phenylindole to facilitate cell identification. Following each incubation step, the samples were rinsed with PBS five times for 5 min each. For each immunofluorescence quantification, we now specify: field size (1260 μm × 1260 μm for 10× images, 630 μm × 630 μm for 20× images, 315 μm × 315 μm for 40× images, microscope model (FV1000, Olympus), and the thresholding method (automatic Otsu thresholding in ImageJ followed by manual verification). Longitudinal sections (10 μm thick) were cut along the long axis of the graft, including the anastomosis (both proximal and distal), the quarter (1/4 length from each anastomosis), and the mid-portion. For CD31 immunofluorescence, images were taken at 20× magnification. The percentage of CD31^+^ area relative to the total luminal surface area was calculated using ImageJ: a region of interest (ROI) was drawn along the luminal border (100 μm width × the width of the graft), and the thresholded CD31^+^ area was divided by the total ROI area. For each region, 5 random fields from 3 independent sections per animal were averaged.

Data were collected from at least three different areas in each group.

### 4.19 Neoarterial adventitia and intima tissue evaluation

For the process of immunofluorescence staining, careful dissection of the adventitia and intima components from the neovascular tissue was performed under a microscope. The tissues were then permeabilized with a 0.5% solution of Triton X-100 and fixed using 4% paraformaldehyde. Following this step, the samples were incubated at 37°C for 2 h in a blocking solution of 5% normal goat serum. The neointima was exposed to a primary antibody mixture comprising CD31 diluted 1:100 (Novus, NB 600-562) and CD93 (1:300 dilution; Abcam), while the neo-adventitia was treated with the CD31 primary antibody at a 1:100 dilution along with the LYVE1 antibody (1:300 dilution, Novus). After the primary incubation period, the samples received fluorescent secondary antibodies: Alexa Fluor 594 IgG and Alexa Fluor 488 IgG, both at a dilution of 1:500. Immunofluorescence images were obtained via laser confocal microscopy (FV 1000, Olympus, Japan) and were further analyzed with ImageJ software to assess cellular distribution and organization of the tissue.

### 4.20 Examination of transmission electron microscopy (TEM)

To explore the ultrastructural properties of neotissue in arterial grafts, transmission electron microscopy (TEM) imaging was carried out with an H7500 microscope from Hitachi (Japan). The arterial grafts were fashioned into 2 mm × 2 mm × 2 mm cubes and treated as follows: the samples underwent three washes in PBS, each lasting 5 min, were then fixed using 2.5% glutaraldehyde, and dehydrated through a sequential series of ethanol concentrations (30%, 50%, 70%, 90%, and 100%). Following this, the samples were infused with epoxy resin. Ultrathin sections were created with an ultramicrotome and stained with 2% uranyl acetate and lead citrate to improve contrast. Images were captured at random locations across the sections to guarantee representative sampling of the grafts.

### 4.21 Isometric force measurement

To assess the vascular reactivity of neo arteries, isometric contraction forces were quantified using an *in vitro* organ bath system, in accordance with the procedure outlined by Liu et al. Neo arterial segments measuring 3 mm in length were positioned between two stainless steel hooks and suspended within a four-chamber organ bath (Radnoti, USA). One hook connected to a force transducer while the other was secured to a stationary plastic support. The transducer interfaced with a PowerLab system (ADInstruments, Australia) for the purpose of real-time data acquisition. The chambers were filled with Krebs buffer, kept at 37°C, and continuously supplied with a mixture of 95% O_2_ and 5% CO_2_ for oxygenation. Following a 30-min equilibration phase, the vascular rings were subjected to stimulation with 60 mM KCl, proceeded by washes until the original resting tension was regained. The arterial rings were then subjected to pre-contraction using 10-6 M phenylephrine (PE), after which concentration-response curves were evaluated with 10-100 mM KCl, 10-10 to 10-4 M PE, and acetylcholine (ACh). The vasodilatory responses were adjusted to PE-induced contractions and expressed as percentages. The capacities for vascular contraction and relaxation were quantified by calculating the area under the concentration-response curve (AUC).

### 4.22 Western-blot analysis

Proteins were isolated from the collected tissues utilizing RIPA lysis buffer (Beyotime, China), which contained a combination of protease and phosphatase inhibitors. Identical volumes of protein samples underwent separation via 10% sodium dodecyl sulfate-polyacrylamide gel electrophoresis (SDS-PAGE) and were subsequently transferred to polyvinylidene fluoride (PVDF) membranes (Millipore, Billerica, MA, USA). Following a blocking step, the membranes were treated with the following primary antibodies: VEGFA (Proteintech, 1:3000, 66828-1-Ig), CD31 (Proteintech, 1:300, 28083-1-AP), CXADR (Santa Cruz Biotechnology, 1:3000, sc-373791), MAPK (Proteintech, 1:3000, 83533-1-RR), TNF-α (Cell Signaling, 1:1000, 8698S), and NF-κB (p56) (Proteintech, 1:3000, 14220-1-AP). This was followed by incubation with the relevant HRP-conjugated secondary antibodies. The expression level of GAPDH (Sangon Biotech, 1:3000, D190090) served as an internal control for reference. Each experiment was conducted in triplicate to ensure consistency in results. The relative intensity was quantified using Image J software.

### 4.23 Proteomics-total protein extraction

Remove the samples from their frozen state and place them on ice. The samples were then mixed with a protein lysis buffer containing 8M urea and 1% SDS, along with the inclusion of a suitable protease inhibitor to curb protease activity. This mixture underwent treatment using a high-flux tissue grinding machine for three intervals of 180 s each. Following this, non-contact cryogenic sonication was applied for a duration of 30 min. After centrifugation at 16,000 g and 8°C for 30 min, the protein concentration in the collected supernatant was assessed using the Bicinchoninic acid (BCA) method with the BCA Protein Assay Kit from Thermo Scientific. The protein quantification process adhered to the instructions provided in the kit. Once the protein quantification was complete, SDS-PAGE electrophoresis was conducted.

### 4.24 Proteomics-protein digestion

100 μg of protein was re-suspended in a Triethylammonium bicarbonate buffer (TEAB) at a final concentration of 100 mM. The solution was then subjected to reduction using Tris(2-carboxyethyl)phosphine (TCEP) at a concentration of 10 mM for 60 min at 37°C, followed by alkylation with iodoacetamide (IAM) at a concentration of 40 mM for 40 min at room temperature in the dark. After spinning at 10,000 g for 20 min at 4°C, the pellet was collected and re-suspended in 100 μL of Triethylammonium bicarbonate buffer (TEAB) at the same final concentration of 100 mM. Trypsin was then introduced at a ratio of 1:50 (trypsin to protein mass) and incubated overnight at 37°C.

### 4.25 Proteomics-peptide desalting and quantification

Following the digestion with trypsin, the resulting peptides were subjected to drying through a vacuum pump. Subsequently, the peptides, which had undergone enzymatic drainage, were re-dissolved in a solution of 0.1% trifluoroacetic acid (TFA). The desaltation process of the peptides utilized HLB, and they were then dried again using a vacuum concentrator. Ultimately, the quantification of the peptides was carried out utilizing the NANO DROP ONE (Thermo Scientific) based on UV absorption measurements.

### 4.26 Proteomics-DIA mass detection

Peptide quantification results were obtained through analysis on a VanquishNeo system paired with a timsTOF HT mass spectrometer (Bruker, Germany) at Majorbio Bio-Pharm Technology Co. Ltd. in Shanghai, China. To summarize, a Homemade column (15 cm × 100 μm, 1.7 μm) was employed along with solvent A (water with 2% ACN and 0.1% formic acid) and solvent B (water with 80% ACN and 0.1% formic acid). The chromatography was conducted over a duration of 8 min. Data-independent acquisition (DIA) was performed using the timsTOF HT mass spectrometer in DIA mode, with detection occurring across a mass range of 70-1050 *m*/*z* for MS1 and 150-2000 *m*/*z* for MS2.

### 4.27 Proteomics-protein identification

Spectronaut software (Version 19) was employed to analyze the DIA raw data. The parameters established were as follows: The range for peptide length was specified between 7 and 52; the enzyme used for cleavage was trypsin/P; a maximum of 2 missed cleavages was permitted; cysteine carbamidomethylation was set as a fixed modification, while methionine oxidation and N-terminal acetylation of proteins were defined as variable modifications; the protein false discovery rate (FDR) threshold was set to ≤ 0.01, the peptide FDR was likewise set to ≤ 0.01, with a Peptide Confidence of >99%, and an XIC width of <75 ppm. The quantification approach for proteins was MaxLFQ.

### 4.28 Proteomics-statistical analyses

The analysis of proteomic data through bioinformatics was conducted utilizing the Majorbio Cloud platform (https://cloud.majorbio.com). The R package “*t*-test” was employed to compute the P-values and Fold Change (FC) for the proteins across the two groups. To identify differentially expressed proteins (DEPs), thresholds were established for fold change (>1.2 or <0.83) and P-value (<0.05). All identified proteins underwent functional annotation via Gene Ontology (GO) (http://geneontology.org) and the KEGG pathway database (http://www.genome.jp/kegg/). The identified DEPs were subsequently analyzed for GO and KEGG enrichment. Additionally, protein-protein interaction assessments were carried out using String v11.5.

### 4.29 Transcriptomics-RNA extraction

Total RNA was extracted using TRIzol Reagent according to the manufacturer's instructions. RNA quality was determined using the NanoDrop 2000 (Thermo Fisher Scientific). Only high-quality RNA (OD260/280 = 1.8∼2.2, OD260/230 ≥ 2.0, RQN≥6.5, 28S:18S ≥ 1.0, >1 μg) was used to construct sequencing library.

### 4.30 Transcriptomics-library preparation and sequencing

RNA extraction and sequencing procedures were conducted at Shanghai Majorbio Bio-Pharm Technology Co. Ltd. (Shanghai, China), adhering to the protocols provided by the manufacturer. In summary, 1 μg of total RNA was utilized to enrich mRNA through polyA selection beads, followed by initial fragmentation of the mRNA. Subsequently, double-stranded cDNA was generated using a cDNA synthesis kit (Invitrogen) along with random primers. The fragmented cDNA underwent end repair, A-tailing, and the process of adapter ligation. The construction of the library was finalized through the PCR amplification of targeted fragments utilizing DNA polymerase. Ultimately, sequencing of the library was executed on the Illumina NovaSeq X Plus platform. The RNA-seq transcriptome library was developed following the SMART-Seq_V4 Ultra Low Input RNA Kit for Sequencing from Clontech (San Diego, CA, USA). To elaborate, the first-strand cDNA was produced using Oligo (dT) primer and MMLV Reverse Transcriptase, which incorporates additional cytosine (C) residues at the 3′ end of the cDNA strand. The second-strand cDNA was formed through a template-switching mechanism. The cDNA underwent amplification via PCR. DNA fragmentation occurred concurrently with the ligation of linkers to the ends of the cDNA fragments. After completing PCR amplification, the library was quantified and sequenced on the Illumina NovaSeq X Plus platform.

### 4.31 Transcriptomics-quality control and mapping to reference genome

Quality control of the raw sequencing data was performed with FastQC software. Reads with low quality (Phred score <20), along with adapter sequences and short reads (length <20 bp), were excluded from the analysis. The filtered clean reads were aligned to the reference genome using either HISAT2 or TopHat 2 software, and the mapping efficiency as well as coverage were assessed to determine the quality of the data.

### 4.32 Transcriptomics-differential expression analysis and functional enrichment

To identify genes that are differentially expressed (DEGs), the expression levels for each gene were determined by employing the reads counts method. The analysis of DEGs was conducted using either DESeq2 or DEGseq software, applying a threshold of FDR 1. Furthermore, KEGG pathway enrichment analysis was conducted to uncover significantly enriched metabolic pathways, with a Bonferroni-corrected significance level of P < 0.05 based on the whole transcriptome background. Additionally, GO functional enrichment analyses were performed utilizing Goatools to annotate the DEGs in terms of biological processes, cellular components, and molecular functions.

### 4.33 Transcriptomics-alternative splice events identification

All alternative splicing events were identified using rMATS or other relevant software based on the mapped reads. The isoforms that were supported by sufficient reads and splice junctions were considered, and the splicing events including exon skipping, alternative 5′ splice site, alternative 3′ splice site, and intron retention were analyzed.

### 4.34 Statistical analysis

All repeated experiments were performed with at least three biological replicates. All analyses were conducted using SPSS23 (IBM). All data are presented as mean ± SD. Comparisons between two groups were determined by Student's t-test, while comparisons among multiple groups were determined by one-way analysis of variance (ANOVA) followed by Tukey's post hoc test. P value of <0.05 was considered statistically significant. Sample sizes were determined based on a power analysis using preliminary patency data. To detect such a difference with α = 0.05 and β = 0.20, a minimum of 5 animals per group was required. For the DMwVG group, we enrolled 18 animals to allow for evaluation at three time points (2, 4, 12 weeks; n = 6 per time point) plus additional animals for mechanistic studies (axitinib, inhibitor injections). Control groups (MwVGs, DES) were evaluated primarily at the 2-week patency endpoint (n = 6 each) because they were expected to have low patency and were not followed long-term.

## Data availability statement

The data that support the findings of this study are available from the corresponding author upon reasonable request.

## Ethics approval and consent to participate

All experimental procedures were approved by the Experimental Animal Welfare and Ethics Committee, the Air Force Military Medical University (approval number: 2023 (kq-065)).

## CRediT authorship contribution statement

**Yidong Hu:** Conceptualization, Data curation, Formal analysis, Investigation, Methodology, Project administration, Resources, Software, Writing – original draft, Writing – review & editing. **Pingping Yuan:** Data curation, Formal analysis, Funding acquisition, Investigation, Methodology, Project administration, Writing – original draft, Writing – review & editing. **Zheqian Zhang:** Investigation, Methodology, Resources, Software, Validation, Visualization. **Gaoyue Yang:** Formal analysis, Funding acquisition, Investigation, Resources, Supervision. **Yuxin Lu:** Formal analysis, Funding acquisition, Investigation, Project administration, Resources. **Xinchi Zhang:** Formal analysis, Methodology, Project administration, Software, Supervision. **Xiaopeng Wu:** Funding acquisition, Investigation, Methodology, Resources, Supervision. **Yajing Zhao:** Funding acquisition, Methodology, Project administration, Resources, Software. **Pengyu Wang:** Funding acquisition, Project administration, Software. **Yujiao Wang:** Formal analysis, Project administration, Resources. **Ning Leng:** Resources, Software, Supervision. **Wenyu Zhan:** Formal analysis, Investigation, Methodology. **Wei Wu:** Conceptualization, Data curation, Funding acquisition, Investigation, Validation, Visualization, Writing – original draft, Writing – review & editing.

## Declaration of competing interest

The authors declare that they have no known competing financial interests or personal relationships that could have appeared to influence the work reported in this paper.

## References

[1] Barnett S.N. (2024). An organotypic atlas of human vascular cells. Nat. Med..

[2] Robinson E. (2025). Pulmonary endarterectomy for chronic thromboembolic pulmonary hypertension secondary to expanded polytetrafluoroethylene valved pulmonary conduit thrombosis. JACC Case Rep..

[3] Park J. (2019). Arteriovenous graft patency outcomes and prognostic factors. Vascular.

[4] Cai Z. (2022). Acellular vascular scaffolds preloaded with heparin and hepatocyte growth factor for small-diameter vascular grafts might inhibit intimal hyperplasia. Cell Transplant..

[5] Bao J. (2015). Hemocompatibility improvement of perfusion-decellularized clinical-scale liver scaffold through heparin immobilization. Sci. Rep..

[6] Brown M., Li J., Moraes C., Tabrizian M., Li-Jessen N.Y.K. (2022). Decellularized extracellular matrix: new promising and challenging biomaterials for regenerative medicine. Biomaterials.

[7] Cheng Q. (2026). Reinforced biotubes as readily available and regenerative vascular grafts. Nat. Commun..

[8] Rickel A.P., Deng X., Engebretson D., Hong Z. (2021). Electrospun nanofiber scaffold for vascular tissue engineering. Mater. Sci. Eng., C.

[9] Anderson A.R. (2025). Engineering the microstructure and spatial bioactivity of MAP scaffolds instructs vasculogenesis *in vitro* and modifies vessel formation *in vivo*. Adv. Funct. Mater..

[10] Ferreira H.P., Moroni L., Bergmeister H., Goncalves I.C. (2026). Engineering antithrombogenic surfaces in synthetic small-diameter vascular grafts: a review of passive strategies. Acta Biomater..

[11] Ding K., Yu X., Wang D., Wang X., Li Q. (2023). Small diameter expanded polytetrafluoroethylene vascular graft with differentiated inner and outer biomacromolecules for collaborative endothelialization, anti-thrombogenicity and anti-inflammation. Colloids Surf. B Biointerfaces.

[12] Zeibi Shirejini S., Carberry J., Alt K., Gregory S.D., Hagemeyer C.E. (2023). Shear-Responsive drug delivery systems in medical devices: focus on thrombosis and bleeding. Adv. Funct. Mater..

[13] Wang Z. (2024). Tailored endothelialization enabled by engineered endothelial cell vesicles accelerates remodeling of small-diameter vascular grafts. Bioact. Mater..

[14] Sanchez P.F., Brey E.M., Briceno J.C. (2018). Endothelialization mechanisms in vascular grafts. J. Tissue Eng. Regen. Med..

[15] Yuan L. (2026). Construction of small-diameter vascular grafts by electrospun zwitterionic diselenide-containing poly(ester urethane)urea with enhanced endothelialization. Acta Biomater..

[16] Fu M. (2025). 3D Micro-Nano hierarchical constructs enabling transmural ingrowth of adventitia improves the “Fallout endothelialization” of vascular grafts. Adv. Funct. Mater..

[17] Zhang Z.Q. (2025). Rapid endothelialization of printed vascular grafts by perivascular niche-circulating endothelial progenitors crosstalk. Nat. Commun..

[18] Mirasadi K. (2026). 4D printing of magnetically responsive shape memory polymers: toward sustainable solutions in soft robotics, wearables, and biomedical devices. Adv. Sci. (Weinh.).

[19] Shi J. (2026). Sacrificial biofabrication for vascularization: concept, materials, technologies, and applications. Adv. Mater..

[20] Liu X. (2025). Spatiotemporally programming microenvironment to recapitulate endochondral ossification via greenhouse-inspired bionic niche. Adv. Mater..

[21] Nguyen H.T. (2023). Engineered vasculature for cancer research and regenerative medicine. Micromachines.

[22] Janssen R. (2025). Biofabrication directions in recapitulating the immune System-on-a-Chip. Adv. Healthcare Mater..

[23] Wang Y. (2023). 3D elastomeric stent functionalized with antioxidative and perivascular tissue regenerative activities ameliorated PVT deprivation-induced vein graft failure. Adv. Healthcare Mater..

[24] Miao S., Sun L., Zhao Y. (2026). Bio-Inspired super-surfaces from frost-assisted freeze-casting strategy. Adv. Mater..

[25] Joukhdar H. (2021). Ice templating soft matter: fundamental principles and fabrication approaches to tailor pore structure and morphology and their biomedical applications. Adv. Mater..

[26] Montini-Ballarin F. (2016). Mechanical behavior of bilayered small-diameter nanofibrous structures as biomimetic vascular grafts. J. Mech. Behav. Biomed. Mater..

[27] Ibrahim A. (2024). Genetic analysis: therapeutic drug monitoring of metformin and glimepiride on diabetic patients' plasma including genetic polymorphism. J. Adv. Pharm. Technol. Res..

[28] Xiao W. (2022). Recombinant DTbeta4-inspired porous 3D vascular graft enhanced antithrombogenicity and recruited circulating CD93(+)/CD34(+) cells for endothelialization. Sci. Adv..

[29] Zhang Y., Lui W.Y. (2023). CXADR: from an essential structural component to a vital signaling mediator in spermatogenesis. Int. J. Mol. Sci..

[30] Angelova D.M. (2025). Single-cell RNA sequencing identifies CXADR as a fate determinant of the placental exchange surface. Nat. Commun..

[31] Rodriguez-Soto M.A. (2024). Redefining vascular repair: revealing cellular responses on PEUU-gelatin electrospun vascular grafts for endothelialization and immune responses on *in vitro* models. Front. Bioeng. Biotechnol..

[32] Han Y. (2023). Cyclic stretch promotes vascular homing of endothelial progenitor cells via Acsl1 regulation of mitochondrial fatty acid oxidation. Proc. Natl. Acad. Sci. U. S. A..

[33] Erkan M.H. (2025). Effect of surface coatings on endothelialization and biofilm in PTFE vascular grafts. Int. J. Artif. Organs.

[34] Park J. (2025). Fully biologic endothelialized-tissue-engineered vascular conduits provide antithrombotic function and graft patency. Cell Stem Cell.

[35] Liu Y. (2023). Multifunctional nanoparticle-VEGF modification for tissue-engineered vascular graft to promote sustained anti-thrombosis and rapid endothelialization. Front. Bioeng. Biotechnol..

[36] Pennel T., Bezuidenhout D., Koehne J., Davies N.H., Zilla P. (2018). Transmural capillary ingrowth is essential for confluent vascular graft healing. Acta Biomater..

[37] Clowes A.W., Kirkman T.R., Reidy M.A. (1986). Mechanisms of arterial graft healing. Rapid transmural capillary ingrowth provides a source of intimal endothelium and smooth muscle in porous PTFE prostheses. Am. J. Pathol..

[38] Yarali E. (2024). 4D printing for biomedical applications. Adv. Mater..

[39] Yousefi M.A. (2026). 4D printing for minimally invasive biomedical applications: programmable smart materials for deployable devices, drug delivery, and tissue regeneration. Mater. Des..

[40] Yousefi M.A., Rahmatabadi D., Baniassadi M., Bodaghi M., Baghani M. (2025). 4D printing of multifunctional and biodegradable PLA-PBAT-Fe(3)O(4) nanocomposites with Supreme Mechanical and shape memory properties. Macromol. Rapid Commun..

[41] Li M. (2024). Metformin syncs CeO(2) to recover Intra- and extra-cellular ROS homeostasis in diabetic wound healing. Small.

[42] Lyle C., McCormick F. (2010). Integrin alphavbeta5 is a primary receptor for adenovirus in CAR-negative cells. Virol. J..

[43] Farmer C., Morton P.E., Snippe M., Santis G., Parsons M. (2009). Coxsackie adenovirus receptor (CAR) regulates integrin function through activation of p44/42 MAPK. Exp. Cell Res..

[44] You Y.Y. (2024). DLK1 promoted ischemic angiogenesis through notch 1 signaling in endothelial progenitor cells. Acta Pharmacol. Sin..

[45] Kansler E.R. (2022). Cytotoxic innate lymphoid cells sense cancer cell-expressed interleukin-15 to suppress human and murine malignancies. Nat. Immunol..

[46] Qiu Y., Zhang C., Zhang G., Tao J. (2018). Endothelial progenitor cells in cardiovascular diseases. Aging Med (Milton).

[47] Salih A.R.C. (2026). Liver tissueoid on-a-Chip modeling liver regeneration and allograft rejection. Adv. Mater..

[48] Asahara T. (1997). Isolation of putative progenitor endothelial cells for angiogenesis. Science.

[49] Bianconi V. (2018). Endothelial and cardiac progenitor cells for cardiovascular repair: a controversial paradigm in cell therapy. Pharmacol. Ther..

[50] Sun Y. (2026). Bilayer vascular grafts separately loaded with sodium copper chlorophyllin and keratin-based hydrogen sulfide donor with pro-endothelialization, anti-thrombogenicity, anti-inflammation, and anti-calcification properties. Bioact. Mater..

[51] Melnik T., Jordan O., Corpataux J.M., Delie F., Saucy F. (2022). Pharmacological prevention of intimal hyperplasia: a state-of-the-art review. Pharmacol. Ther..

[52] Szafron J.M., Heng E.E., Boyd J., Humphrey J.D., Marsden A.L. (2024). Hemodynamics and Wall mechanics of vascular graft failure. Arterioscler. Thromb. Vasc. Biol..

[53] Breuer T., Jimenez M., Humphrey J.D., Shinoka T., Breuer C.K. (2023). Tissue engineering of vascular grafts: a case report from bench to bedside and back. Arterioscler. Thromb. Vasc. Biol..

[54] Li Y. (2017). Netrin-1 promotes inflammation resolution to achieve endothelialization of small-diameter tissue engineering blood vessels by improving endothelial progenitor cells function in situ. Adv. Sci. (Weinh.).

[55] Augustin H.G., Koh G.Y. (2024). A systems view of the vascular endothelium in health and disease. Cell.

[56] Elliott M.B. (2022). Off-the-shelf, heparinized small diameter vascular graft limits acute thrombogenicity in a porcine model. Acta Biomater..

[57] Fang S. (2021). Small diameter polycaprolactone vascular grafts are patent in sheep carotid bypass but require antithrombotic therapy. Regen. Med..

