# Supplementary material for: Perivascular administration of metformin through 3D minichannels improved adventitia ingrowth and endothelialization for electrospinning vascular grafts
Source: Bioact Mater. 2026 Jul 23;66:976–97. doi: 10.1016/j.bioactmat.2026.07.027 (PMC13425663; doi:10.1016/j.bioactmat.2026.07.027)
Supplement: Multimedia component 1 [file mmc1.docx]

**Perivascular Administration of Metformin Through 3D Minichannels Improved Adventitia Ingrowth and Endothelialization for Electrospinning Vascular Grafts**

**Supporting Information**


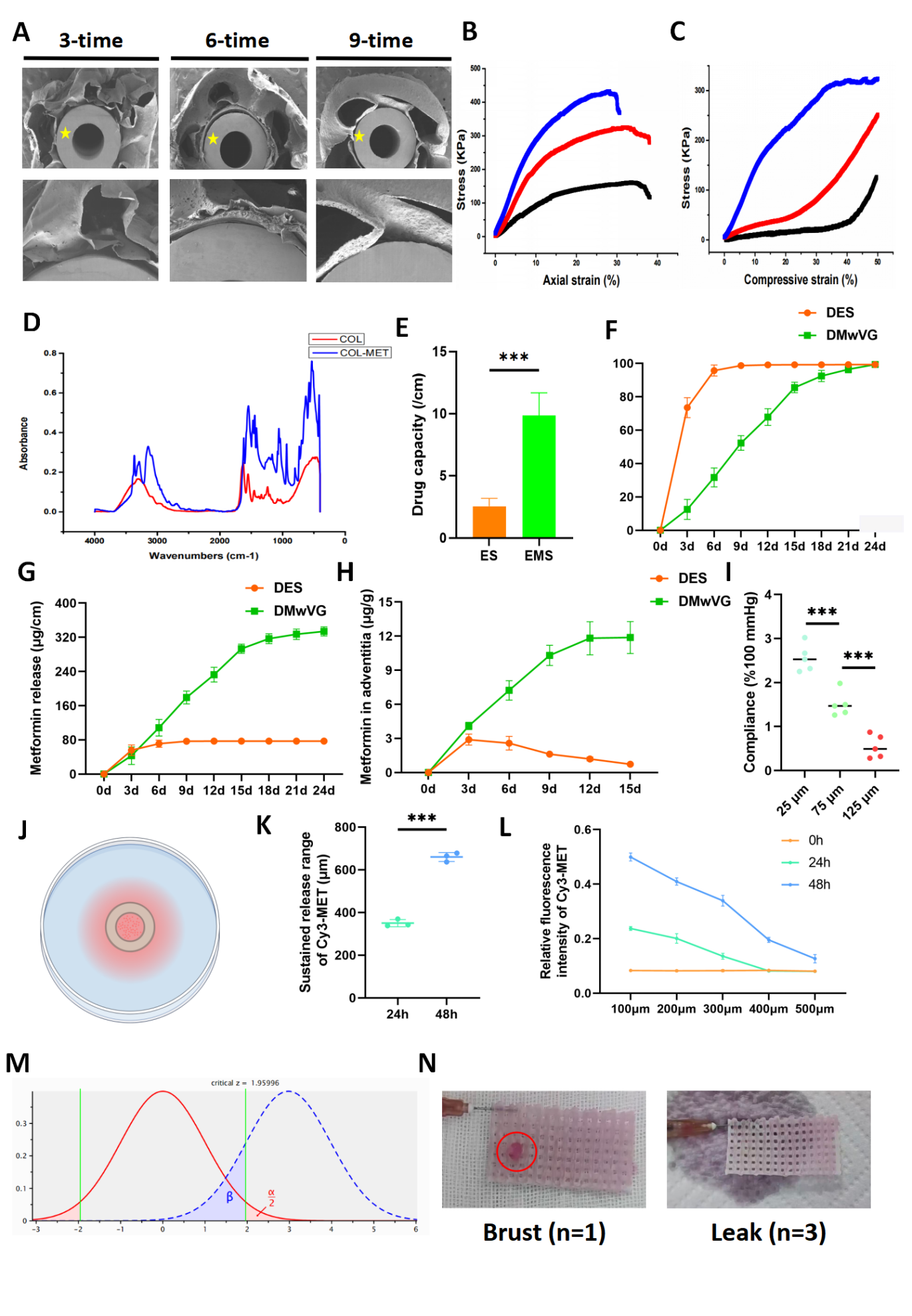


**Figure S1.** **The mechanical properties and drug-loading characteristics of MS.** (A) Scanning electron microscopy images of MS with different coating times, with yellow stars indicating silicone tubes used for support. (B-C) Stress-strain curves for compression and tension of MS with varying coating times. (D) Representative Micro-FTIR spectra of the PBS-loaded MwVGs and metformin-loaded MwVGs. (E) Drug capacity of metformin from DES and DMwVGs. (F) Drug release profile of metformin from DES and DMwVGs within 24 days. (G) The absolute cumulative release of metformin at each time point (n=3 independent samples). (H) Quantity of metformin in adventitia at each time point (n=3 independent samples). (I). Compliance of 25μm, 75μm and 125μm electrospinning layers (n=5 independent samples). (J) Schematic diagram illustrating the sustained release of Cy3-MET in the matrix gel. (K and L) Distribution of Cy-3 in microchannels within the matrix gel over 24 and 48 hours, respectively (n=3 independent samples). For (E and K), significance was determined by Student’s t-test. For (I), significance was determined by one-way ANOVA followed by Tukey’s post hoc analysis. Plotted data represent means ± SD. **: P<0.01, ***: P<0.001.


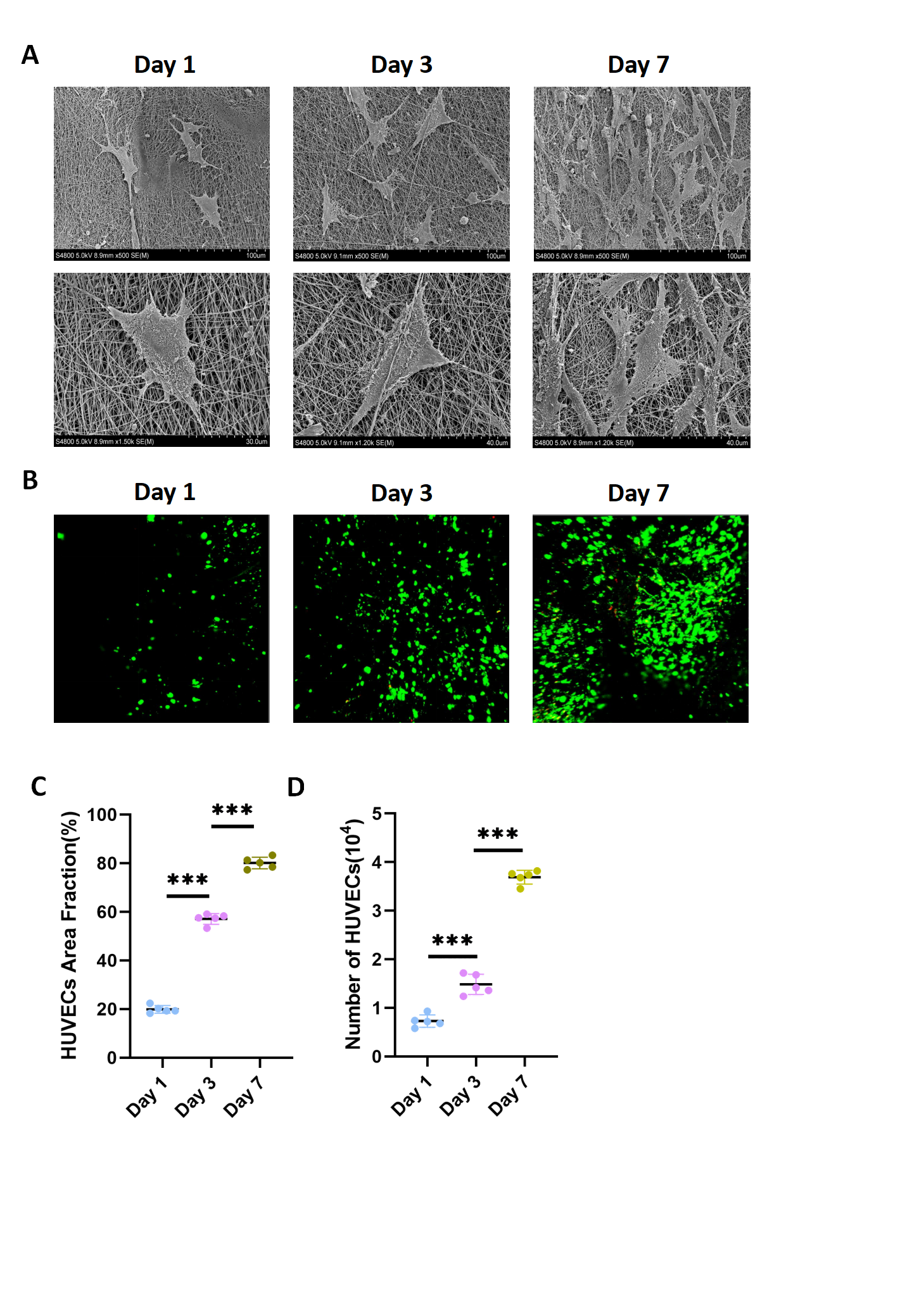


**Figure S2. The cytocompatibility of DMwVGs.** (A) Scanning electron microscopy images illustrate the morphology of HUVECs cultured on DMwVGs surfaces for durations of 1, 3, and 7 days. (B) Live/dead staining was employed to evaluate the viability of HUVECs on DMwVGs surfaces over the same time periods. (C-D) Quantitative analyses were conducted to assess HUVECs coverage and cell counts (n = 5 independent samples). For (C and D), significance was determined by one-way ANOVA followed by Tukey’s post hoc analysis.


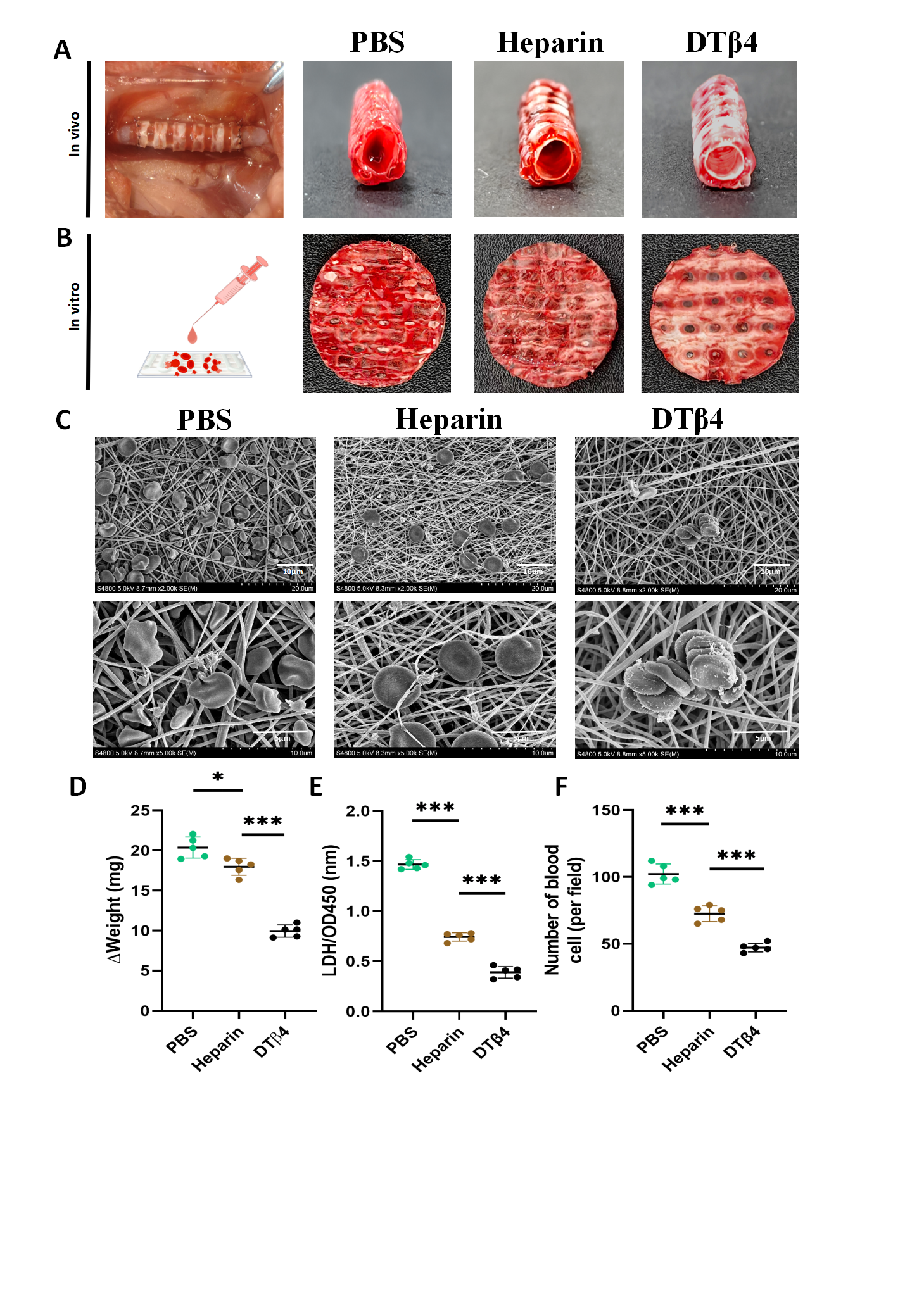


**Figure S3.** **Hemocompatibility of DMwVGs *in vivo* and *in vitro*.** (A) Cross-sectional images illustrating the different grafts exposed to carotid circulation for 2 hours (n = 5 in each group). (B) Gross views of grafts after incubation with recalcified whole blood for 2 hours (n = 5 in each group). (C) The morphology of the incubation of different grafts with platelets and recalcified whole blood was analyzed using SEM (n = 5 independent samples). (D and E) Quantitative analysis of pre- and post-mass weight and LDH assays (n = 5 independent samples). (F) The number of blood cells in different groups. For (D-F), significance was determined by one-way ANOVA followed by Tukey’s post hoc analysis. Plotted data represent means ± SD. *: P<0.05, ***: P<0.001.


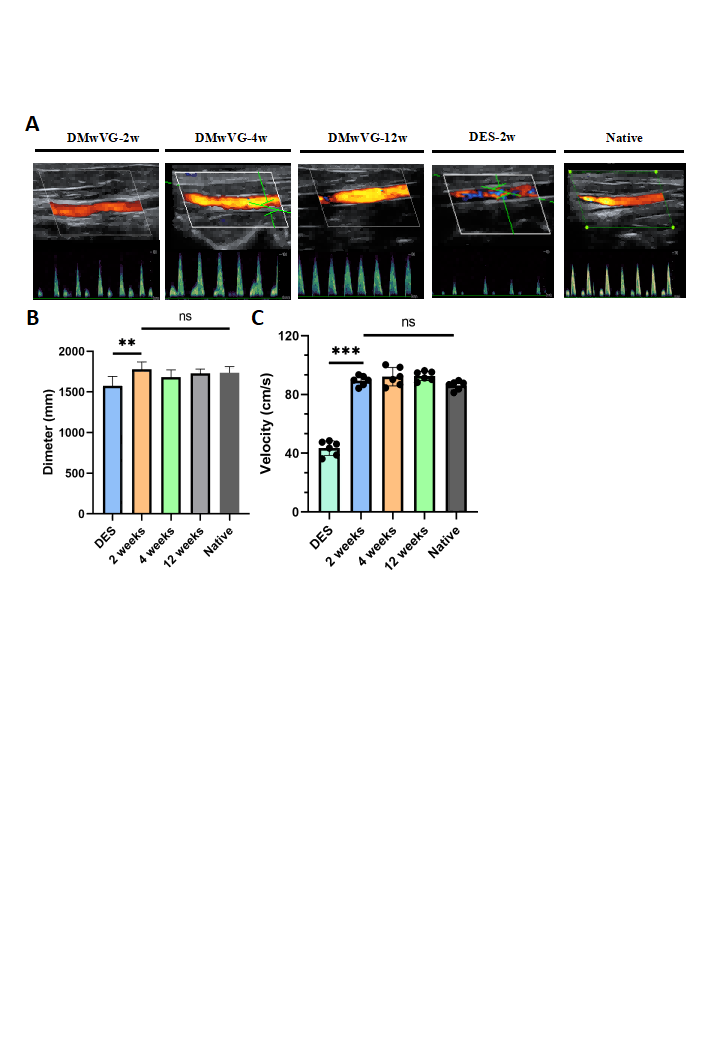


**Figure S4. Hemodynamic function in arteries.** (A) Representative Doppler ultrasound images of the grafts. (B) Average inner diameter of MwVGs and native carotid arteries (n = 6 independent samples). (C) Peak blood flow velocity at each time point (n = 6 independent samples). For (B and C), statistical significance was determined using one-way ANOVA followed by Tukey’s post hoc analysis.


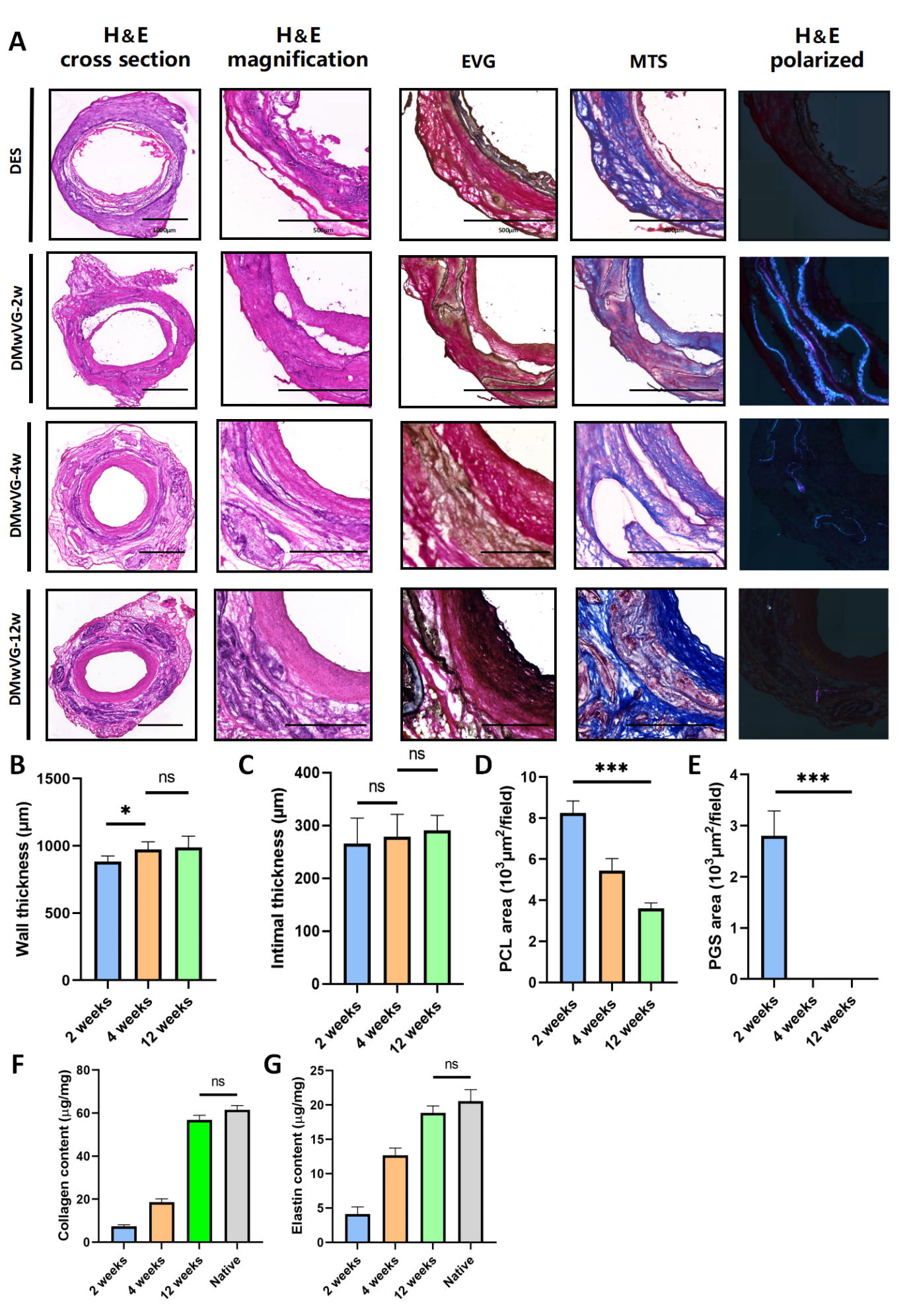


**Figure S5.** **Examining material degradation and host remodeling for DMwVGs.** (A) H&E, MTS and EVG staining of DMwVGs. (B) Wall thickness of neoarteries (n = 5 independent samples). (C) Intimal thickness of neoarteries (n =5 independent samples). (D-E) Quantitative assessment of polymer residuals through 2, 4, and 12 weeks (n = 5 independent samples). (F-G) Quantitative assessment of collagen and elastin content of neoarteries (n = 5 independent samples). For (B-G), significance was determined by one-way ANOVA followed by Tukey’s post hoc analysis. ns: P>0.05, *: P<0.05, ***: P<0.001.


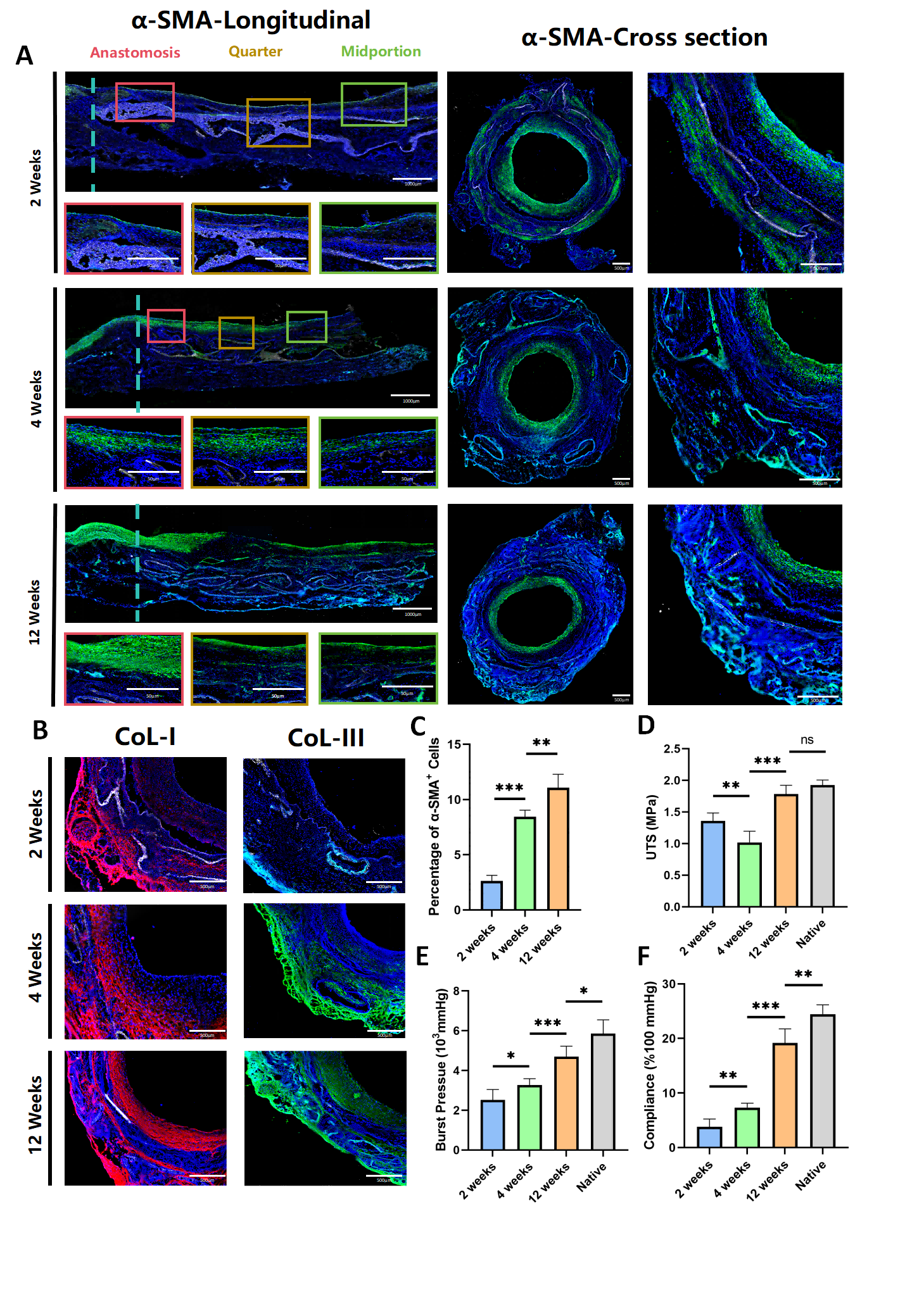


**Figure S6.** **Immunofluorescent analysis of cellular infiltration and ECM remodeling for grafts remodeled through 2, 4, and 12 weeks.** (A) α-SMA immunofluorescence images of longitudinal and cross sections in neoarteries. (B) Col-I and Col-III immunofluorescence images of sections in neoarteries (n = 5 independent samples). (C) Percentage of α-SMA^+^ cells at the middle sites of DMwVGs was quantified at each time point (n = 5 independent samples). (D-F) Mechanical evaluation of the neoarteries. Plotted data represent means ± SD. For (C-F), significance was determined by one-way ANOVA followed by Tukey’s post hoc analysis. ns: P>0.05, *: P<0.05, **: P<0.01, ***: P<0.001.


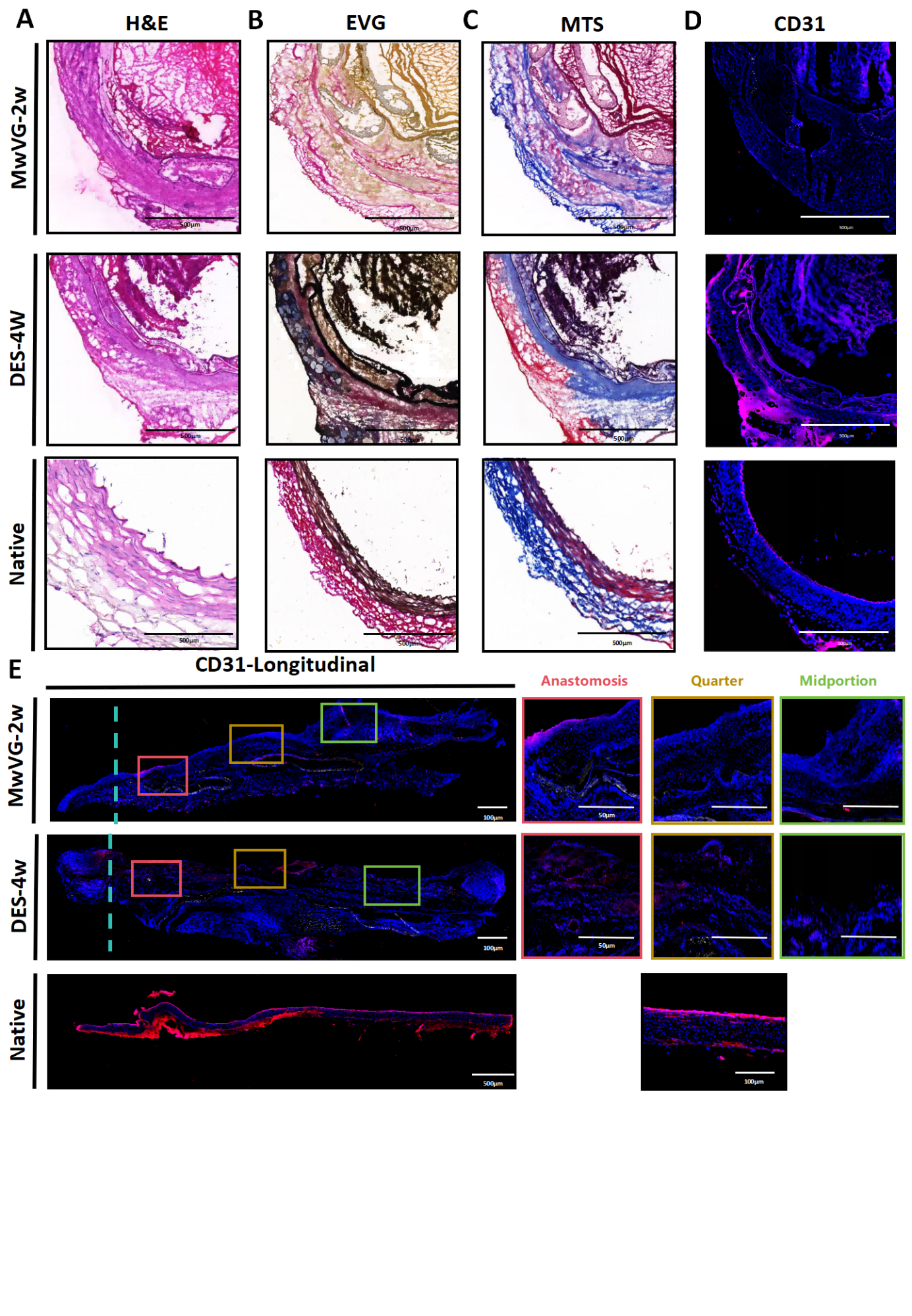


**Figure S7. Cross and longitudinal section views of MwVGs at 2 weeks, DES at 4 weeks and native arteries.** (A) H&E staining of cross sections for arteries. (B-C) Masson and EVG staining of cross sections for arteries. (D-E) Immunofluorescent staining for CD31^+^ cells on cross and longitudinal sections of arteries.


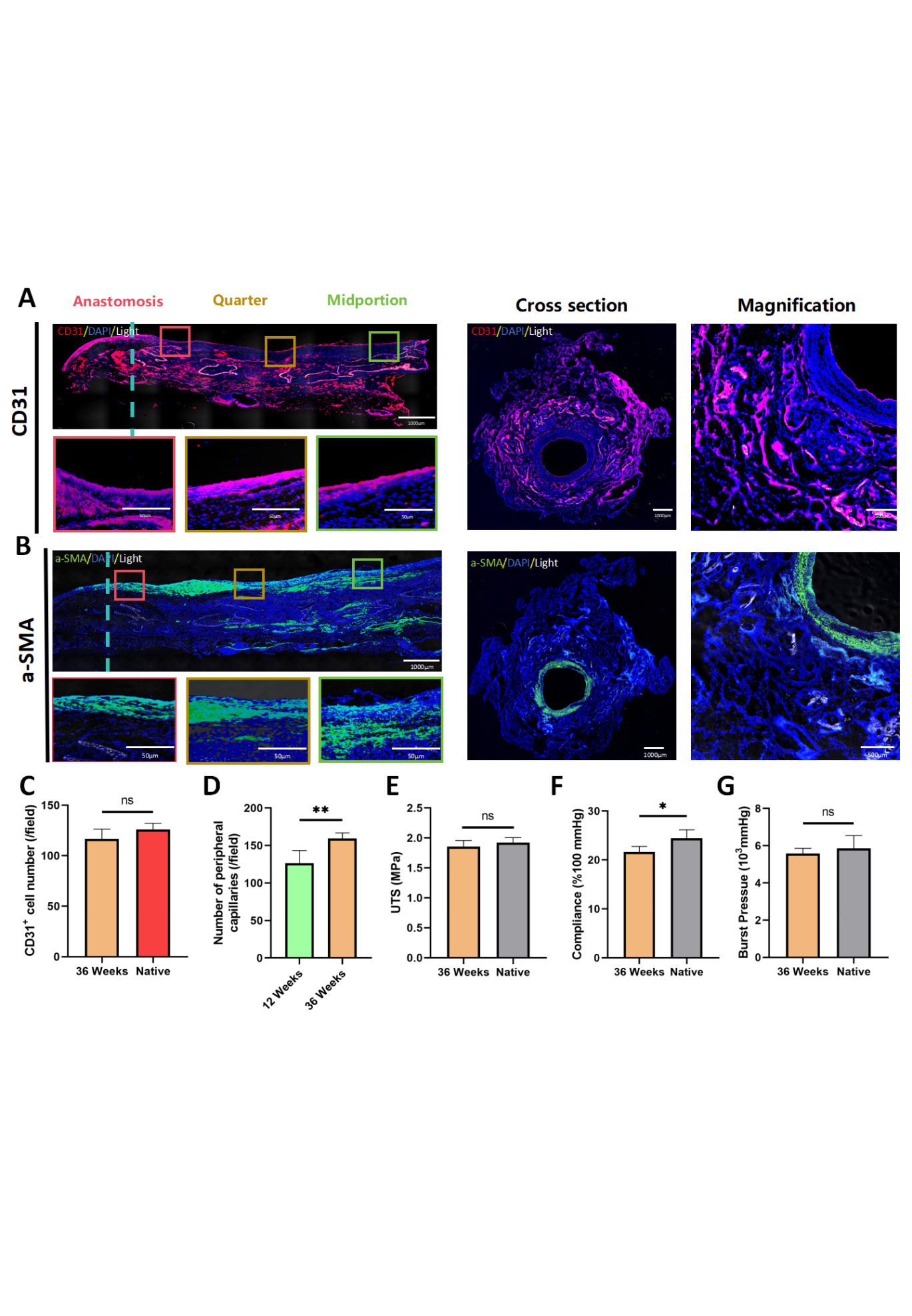


**Figure S8.** **Immunofluorescent analysis of CD31^+^ cells and ECM remodeling in neoarteries at 36 weeks.** (A) Cross-sectional and longitudinal analysis of adventitia angiogenesis and luminal endothelialization in neoarteries at 36 weeks. (B) α-SMA immunofluorescence images of longitudinal and cross sections in neoarteries at 36 weeks. (C) Quantitative analysis of CD31^+^ cells in neoarteries at 36 weeks (n = 6 in each group). (D) Quantitative analysis of peripheral capillaries in neoarteries at 36 weeks (n = 6 in each group). (E) Burst pressure, (F) Compliance, and (G) Ultimate tensile strength of neoarteries at 36 weeks compared to native arteries (n = 6 in each group). For (C-G), significance was determined by Student’s t-test. ns: P>0.05, *: P<0.05, **: P<0.01.


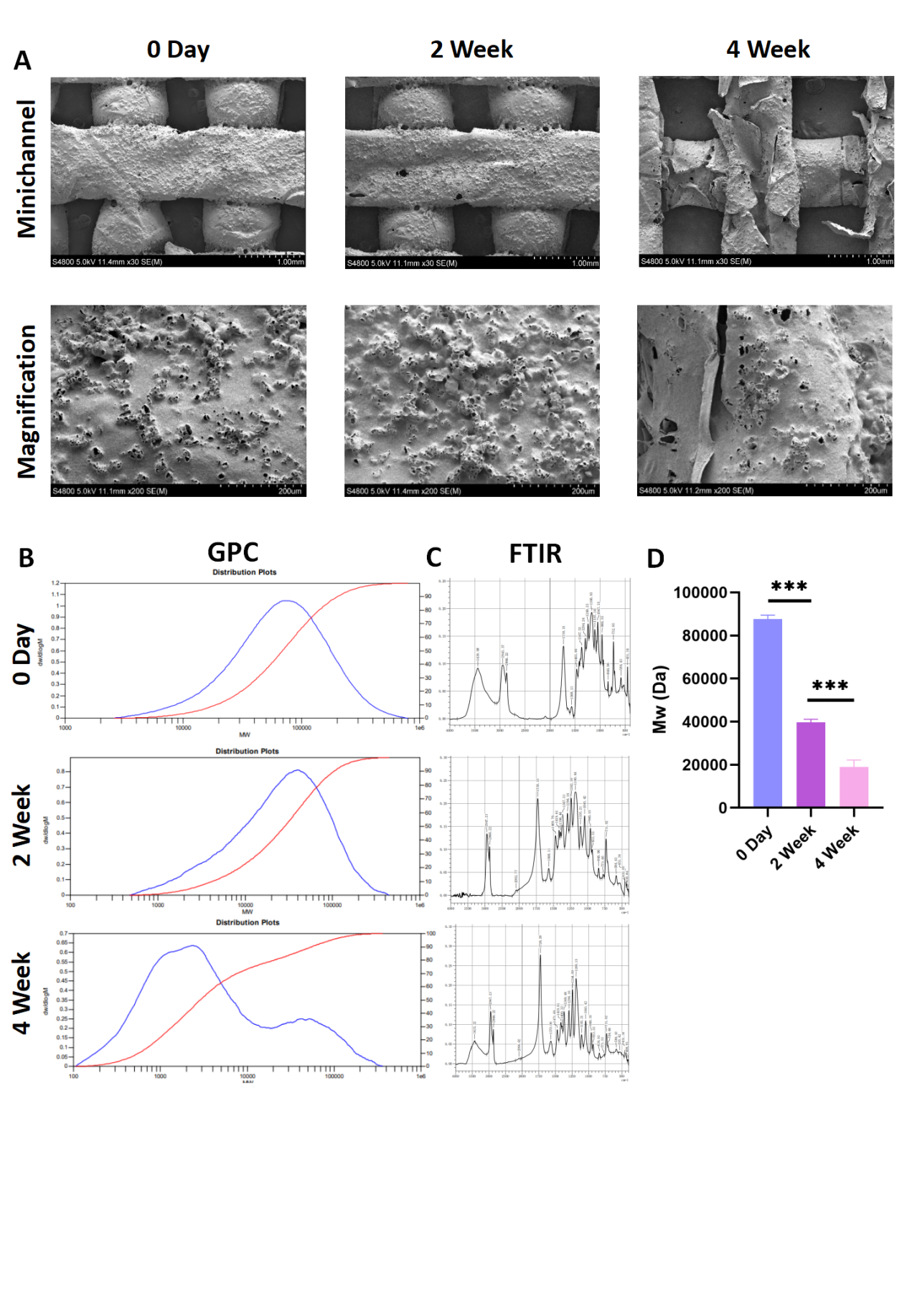


**Figure S9. *In vitro* degradation performance of 3D minichannel scaffold.** (A) SEM images of channel wall at day 0, week 2, and week 4. (B) GPC of 3D minichannel scaffold at each time point (n = 3 in each group). (C) FTIR of 3D minichannel scaffold at each time point. (D) Mw of 3D minichannel scaffold at each time point (n = 3 in each group). For (D), significance was determined by one-way ANOVA followed by Tukey’s post hoc analysis. ns: P>0.05, *: P<0.05, **: P<0.01, ***: P<0.001.

| Abbreviation | Minichannel | Collagen matrix | Metformin | Electrospun PCL layer | DTβ4 coating |
| --- | --- | --- | --- | --- | --- |
| MS | Yes | No | No | No | No |
| ES | No | No | No | Yes | Yes |
| DES | No | No | Yes (in PCL fibers) | Yes | Yes |
| MwVGs | Yes | Yes (PBS) | No | Yes | Yes |
| DMwVGs | Yes | Yes (collagen + metformin) | Yes | Yes | Yes |

**Table S1. Abbreviation, structure and drug loading of each group.**

| Parameter | Measurement Method | n | Value / Result |
| --- | --- | --- | --- |
| Continuity rate | Dye perfusion assay | 20 grafts per batch, 3 batches | 100% |
| Batch failure rate | Perfusion test | 60 grafts total (3 batches) | 6.3% (4/60) |

**Table S2.** **Continuity rate and batch failure rate of the 3D minichannels.**

| Group | n | Pearson r | R^2^ | P-value | Regression equation | Interpretation |
| --- | --- | --- | --- | --- | --- | --- |
| DES | 15 | -0.765 | 0.585 | 0.0009 | Y = -0.1219X + 10.7754 | **Negative correlation:** burst release followed by clearance |
| DMwVG | 15 | 0.924 | 0.854 | <0.0001 | Y = 0.0406X + 1.3512 | **Positive correlation:** sustained release with local retention |
| Combined | 30 | 0.949 | 0.900 | <0.0001 | Y = 0.0387X + 0.2932 | Overall **strong predictive correlation** |

**Table S3. *In vitro*-*in vivo* correlation (IVIVC) analysis for DES and DMwVG groups.**
